# SLFN5 restrains type I interferon responses and promotes glioblastoma via its N-terminal Schlafen core domain

**DOI:** 10.1038/s42003-026-10378-7

**Published:** 2026-05-27

**Authors:** Ricardo E. Perez, Kaitlyn E. Kellar, Niyati Hansaria, Masha Kocherginsky, Mariafausta Fischietti, Frank Eckerdt, Leonidas C. Platanias

**Affiliations:** 1 Robert H Lurie Comprehensive Cancer Center of Northwestern University, Chicago, IL USA; 2 Division of Hematology/Oncology, Department of Medicine, Feinberg School of Medicine, Northwestern University, Chicago, IL USA; 3 Department of Preventive Medicine – Biostatistics & Informatics, Feinberg School of Medicine, Northwestern University, Chicago, IL USA; 4 Department of Medicine, Jesse Brown Veterans Affairs Medical Center, Chicago, IL USA

**Keywords:** Transcriptional regulatory elements, CNS cancer

## Abstract

Schlafen 5 (SLFN5) is an important regulator of Type I interferon (IFN-I) signaling and tumorigenesis, yet the structural basis for its biologic activity remains unclear. Here, we dissect functional domains of SLFN5 and uncover a key role for its N-terminal Schlafen core domain in IFN-stimulated gene (ISG) expression and glioblastoma proliferation, independently of the SWAVDL motif and the C-terminal Walker A/B motifs. Partial deletions within the core domain or substitution of two arginine residues (R271E/R326E) near the zinc finger motif disrupt chromatin association and phenocopy *SLFN5* loss, including de-repression of ISGs that act as immune checkpoints, such as PD-L1 and PD-L2. In contrast, the C-terminal ATPase/helicase activity is dispensable for transcriptional repression and tumor-promoting functions. Structural comparison of human and mouse SLFN5 revealed a conserved architecture, and experimental evidence demonstrates that murine SLFN5 is a functional ortholog of human SLFN5. These findings establish SLFN5 as a key transcriptional modulator of malignant glioblastoma growth by dampening IFN-I responses and have implications for the development of future immunotherapy approaches.

## Abstract (web summary)

This study defines the structural basis of human SLFN5 function by identifying its N-terminal Schlafen core domain as the critical determinant of transcriptional repression of interferon-stimulated genes and glioblastoma growth. It functionally characterized key residues required for this activity, which occurs independently of the C-terminal helicase domain. These findings provide insight into SLFN5-mediated regulation of immune-responsive genes and highlight its potential relevance for therapeutic targeting in brain cancer

## Introduction

Survival rates for patients with glioblastoma (GBM) remain among the lowest across all cancer types^1^ and advanced immunotherapeutic strategies have thus far failed to produce meaningful survival benefits for GBM patients^2,3^. It is well established that the cytokine family of interferons (IFNs) can stimulate anti-tumor responses and enhance tumor susceptibility to immune checkpoint inhibitors (ICIs) via transcriptional regulation of a defined subset of interferon-stimulated genes (ISGs)^4^. Previous work from our laboratory has shown that Type I IFNs (IFN-I) stimulate the expression of Schlafen (SLFN) genes and led to the classification of *SLFN*s as ISGs^5–12^. Other studies demonstrated that the members of this family of genes contribute to the pathophysiology of human diseases by regulating key biological processes, including cellular proliferation, immune responses, chemosensitivity, and DNA replication^10,13–15^.

SLFNs were first identified in mouse thymocytes and were shown to be essential for thymocyte maturation^16^. Since their identification, nine mouse *Slfn* genes (*Slfn1, 2, 3, 4, 5, 8, 9, pseudogene Slfn10, and 14*) located on chromosome 11 and six human *SLFN* genes (*SLFN5, 11, 12, 12 L, 13, and 14*) found on chromosome 17 have been described^12^, highlighting interspecies variability. SLFNs are classified into three subgroups based on size and functional domains. All SLFNs share an N-terminal Schlafen core domain, which includes an endoribonuclease domain and a conserved SLFN sequence termed the “Schlafen box”, whose biological relevance remains unclear^12,17,18^. Subgroup II and III SLFNs additionally contain a linker domain exhibiting the characteristic SWAVDL motif. Only subgroup III SLFNs possess an additional third domain with DNA/RNA helicase activity, making them the longest SLFN subgroup^12,17,18^. Humans express subgroups II and III SLFNs, whereas mice exhibit all three SLFN subgroups. Subgroups I and II SLFNs are predominately located to the cytoplasm, while subgroup III SLFNs can occupy both the cytoplasm and nucleus. Phylogenic analysis suggests that mouse SLFN5 (mSLFN5) and SLFN14 proteins are orthologs for human SLFN5 (hSLFN5) and SLFN14^13,17,19^, but studies confirming overlapping orthological functions are lacking.

SLFN5, a subgroup III SLFN, is predominately located in the nucleus where it acts as a regulator of events that control chromatin structure and transcription. For instance, SLFN5 represses human immunodeficiency virus-1 (HIV-1) transcription by coordinating the epigenetic machinery of cells^20^. Also, SLFN5 suppresses transcription of Zinc Finger E-box Binding Homeobox 1 (ZEB1) in breast cancer cells, with critical consequences for phosphatase and tensin homolog (PTEN) signaling and epithelial-mesenchymal transition (EMT)^21,22^. Work from our group has provided additional evidence for SLFN5 regulating transcriptional events in solid tumors through distinct mechanisms, such as regulation of the activity of the Signal Transducer and Activator of Transcription 1 (STAT1) in glioblastoma (GBM) and the E2F Transcription Factor 7 (E2F7) in pancreatic ductal adenocarcinoma (PDAC)^5,23^. Notably, in both cancers, high SLFN5 expression is associated with poor patient outcomes^5,23^. Importantly, while itself an ISG, SLFN5 participates in a negative feedback loop by restricting ISG transcription in response to IFN-I^5,8^. Given its regulatory role on IFN-I signaling, we have proposed the term “intracellular immune checkpoint” for SLFN5^12,24^.

The functional relevance of distinct SLFN domains remains a topic of ongoing debate. For instance, the biological roles of SLFN box and SWAVDL motif remain largely unexplored. Additionally, while the N-terminal tRNA/rRNA endoribonuclease activity within the SLFN core domain is essential for most SLFN proteins to degrade rRNAs or tRNAs^25–28^, human SLFN5 lacks this enzymatic activity^26^. This distinguishes SLFN5 as an atypical subgroup III SLFN family member and raises questions about the functional relevance of its SLFN core domain. As human SLFN5 is linked to tumorigenesis and controls key transcriptional events^5,12,23^, it is imperative to better understand the contribution and relevance of distinct domain-specific functions. In this study, we employed a systematic mutagenesis approach to identify SLFN5 domains and residues essential for mediating transcriptional responses and malignant GBM cell proliferation. Using a panel of SLFN5 deletion constructs, we define regions within SLFN5 required for ISG suppression and malignant cell proliferation. Targeted mutagenesis further revealed that Arg-271 (R271) and Arg-326 (R326) play essential roles in ISG suppression and GBM proliferation, consistent with their predicted importance for DNA binding^29^. These findings define key functional elements within SLFN5 that are critical for its ability to modulate immune gene expression and drive malignant cell growth. We also provide evidence for functional overlap between human and murine SLFN5, supporting the translational relevance of syngeneic mouse models for future preclinical studies of SLFN5 in immunocompetent settings.

## Results

### Elements within the C-terminal domain are required for SLFN5 nuclear localization in GBM cells

**Fig. 1:**
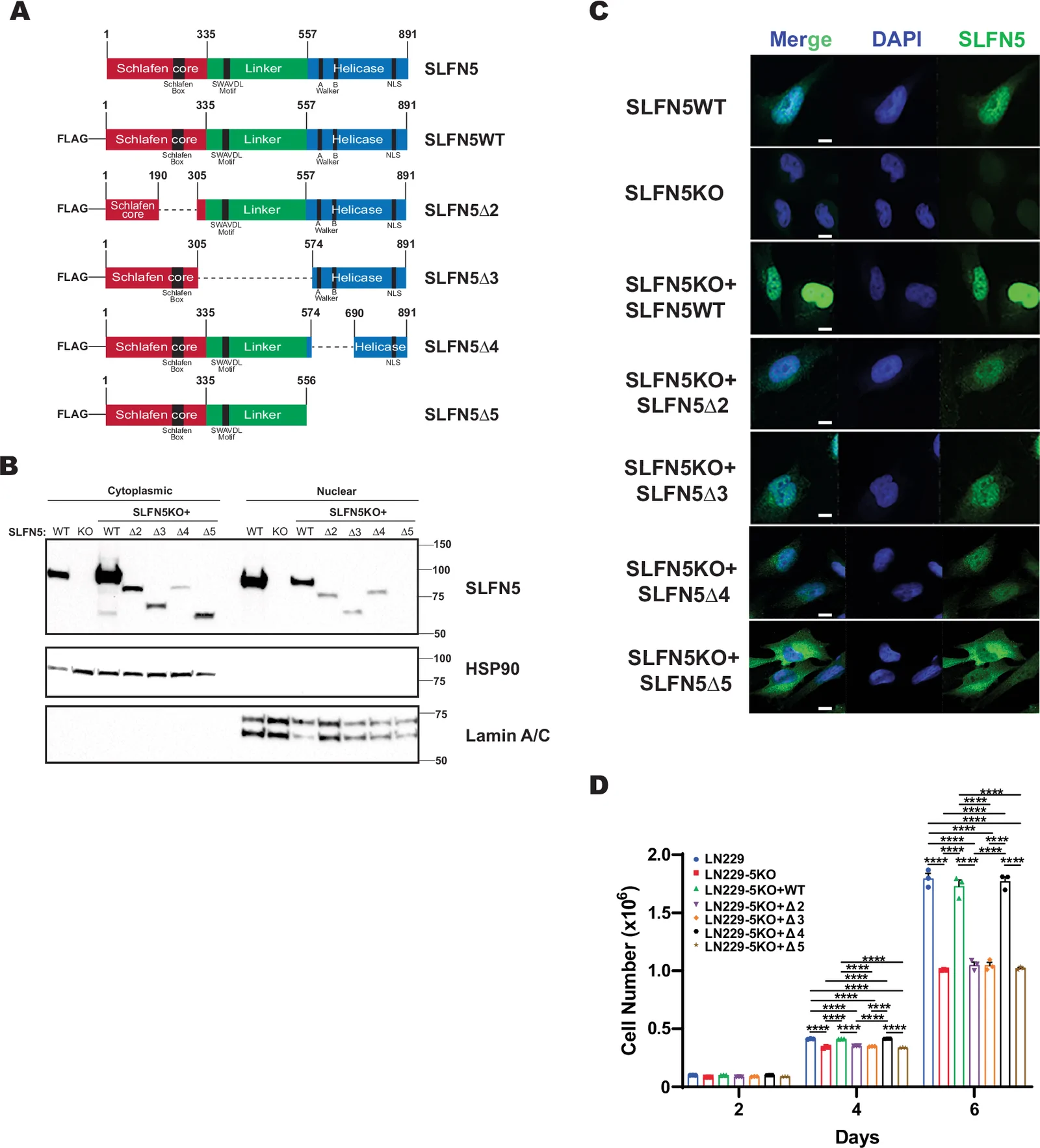
Domain mapping for SLFN5 localization and malignant GBM cell proliferation. **A** Schematic of SLFN5 domain architecture and SLFN5 deletion constructs generated for this study. Schlafen core, Schlafen core domain; Linker, linker domain; Helicase, helicase domain. **B** LN229, LN229-SLFN5KO, and LN229-SLFN5KO cells stably re-expressing the indicated SLFN5 deletion constructs were fractionated into cytoplasmic and nuclear compartments and subjected to immunoblotting with an anti-SLFN5 antibody. Antibodies for the cytoplasmic marker HSP90 and the nuclear marker Lamin A/C were used as controls. **C** Immunofluorescence analysis of LN229 cells expressing the indicated SLFN5 constructs. Cells were stained for DNA (blue) and with anti-SLFN5 antibody (green) to visualize subcellular localization of indicated SLFN5 constructs. Scale bar, 10 μm. **D** LN229, LN229-SLFN5KO, and LN229-SLFN5KO cells stably re-expressing SLFN5 deletion constructs were seeded in 6-well plates (50,000 cells per well), followed by cell counting on days 2, 4, and 6 to assess GBM cellular proliferation. Data represent means ± SEM of three independent experiments, each done in three replicates. Statistical analysis was performed using ordinary two-way analysis of variance (ANOVA) followed by Tukey’s multiple comparisons test. The outcomes were log2-transformed to satisfy the normality assumption. ****, *p* < 0.0001.

Our previous work established that SLFN5 attenuates ISG expression in response to IFN-I treatment in U87 GBM cells^5^. To better understand which domains are important for this repressive transcriptional function, we generated several SLFN5 deletion constructs assigned as SLFN5WT (1–891), SLFN5Δ2 (1–190,305–891) lacking a portion of the Schlafen core domain including the “Schlafen box”; SLFN5Δ3 (1–305,574–891) lacking a small portion of the Schlafen core and the entire linker domain including the conserved SWAVDL motif; SLFN5Δ4 (1–574,690–891) lacking a portion of the helicase domain including Walker A/B motifs; and SLFN5Δ5 (1–556) lacking the helicase domain, including the nuclear localization signal (NLS). In initial experiments, we re-expressed these deletion constructs into LN229-SLFN5KO cells (Fig. 1A) and examined the subcellular localization of these deletions compared to that of wild-type SLFN5 that was previously shown to have a primarily nuclear localization^20,30^. All SLFN5 deletion constructs with the intact C-terminal helicase domain were detected in the cytoplasm and the nucleus by immunoblotting following cellular fractionation (Fig. 1B). SLFN5WT, SLFN5Δ2, SLFN5Δ3, and SLFN5Δ4 were also found to be predominately expressed in the nucleus according to immunofluorescence staining (Fig. 1C). Interestingly, the SLFN5Δ5 deletion lacking the C-terminal helicase domain was exclusively cytoplasmic, likely because this construct lacks the functional NLS (Figs. 1B and 1C), which is essential for proper nuclear SLFN5 localization^20,26^.

### Distinct SLFN5 domain elements promote proliferation independent of helicase Walker motifs

Previous studies have demonstrated regulatory effects for SLFN5 on cellular proliferation of different types of malignant cells, including GBM, pancreatic adenocarcinoma, and breast cancer^5,21,23^. Notably, in both GBM and pancreatic cancer cells, loss of SLFN5 led to a slower proliferation rate as compared to cells expressing SLFN5^5,23^. To investigate the functional relevance of distinct SLFN5 domains, we performed cell proliferation assays using LN229-SLFN5KO cells, in which the different SLFN5 deletion constructs were ectopically expressed. Re-expression of SLFN5WT and SLFN5Δ4, a mutant that lacks the Walker A and B motifs within the C-terminal helicase domain, restored growth kinetics comparable to those of parental LN229 cells (Fig. 1D). In contrast, the growth patterns of cells in which SLFN5Δ2, SLFN5Δ3, and SLFN5Δ5 were re-expressed closely resembled those of SLFN5KO cells with significantly slower proliferation compared to parental LN229 cells or LN229-SLFN5KO complemented with SLFN5WT (Fig. 1D). These findings raise the possibility that SLFN5 nuclear localization is critical for its effects on cellular proliferation. Furthermore, while the Walker A and B motifs appear to be dispensable for supporting proliferation, motifs lacking in SLFN5Δ2 (aa 191-304) and motifs absent in SLFN5Δ3 (aa 306–573) seem to be required for cancer cell proliferation.

### Shared SLFN5 domain requirements for GBM cell proliferation and repression of IFNα-mediated ISG expression

**Fig. 2:**
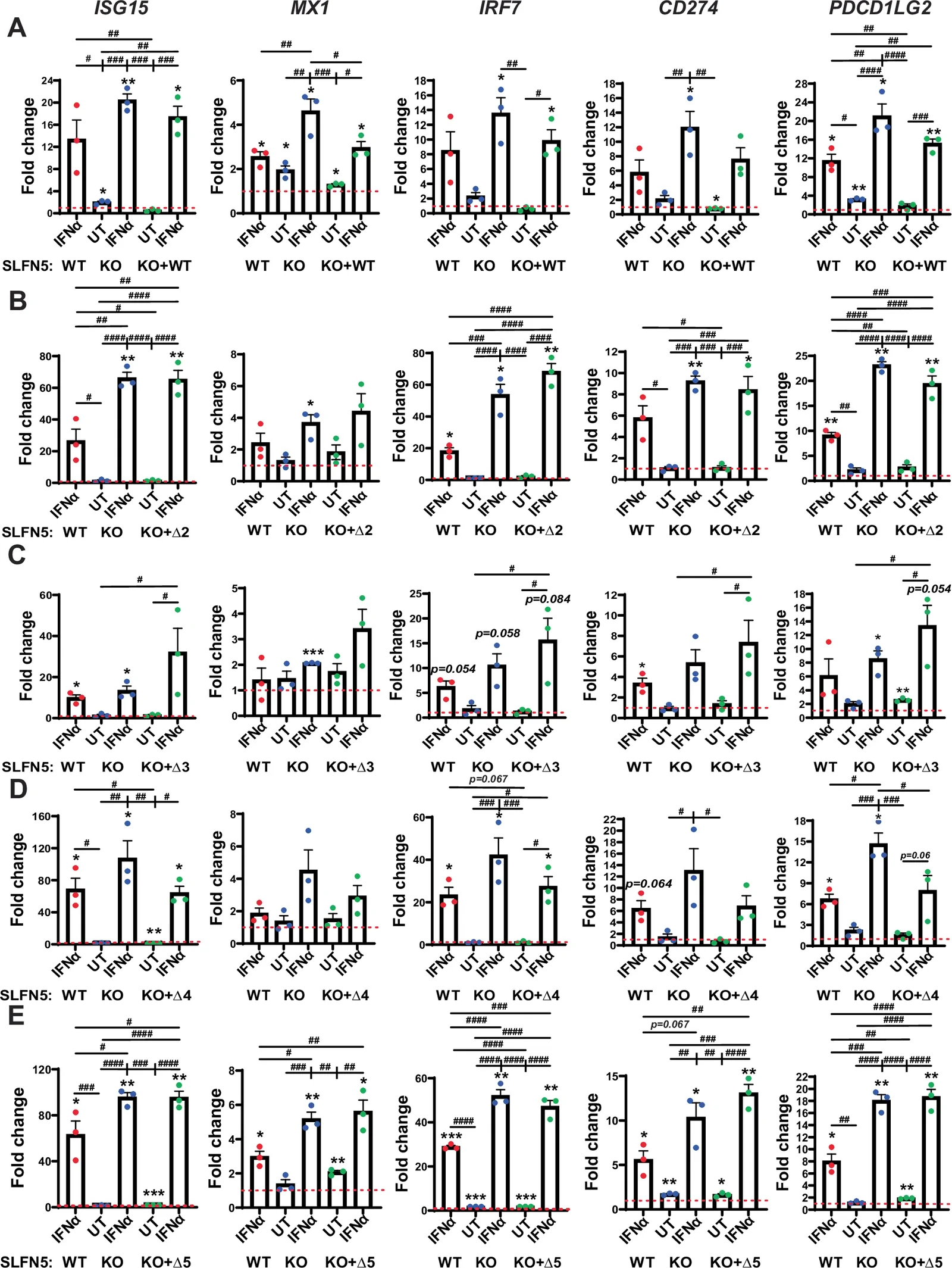
Mapping of SLFN5 domain-specific requirements for ISG repression. **A**–**E** LN229 (WT), LN229-SLFN5KO (KO) and LN229-SLFN5KO cells re-expressing the indicated SLFN5 deletion mutants were serum starved for 24 hours and then treated with 5000 IU/mL IFNα for 6 hours. mRNA expression of *ISG15*, *MX1*, *IRF7*, *CD274*, and *PDCD1LG2* was assessed by quantitative RT-PCR using *GAPDH* for normalization. Data are expressed as fold change relative mRNA expression of indicated genes over LN229-WT untreated cells (red dashed line) and represent means ± SEM of three independent experiments, each done in three replicates. Statistical analyses were performed using One sample *t* test to compare average fold change to FC = 1 (untreated LN229-WT). *, *p* < 0.05; **, *p* < 0.01; and ***, *p* < 0.001. In addition, statistical significance was assessed between pairs of experimental conditions (e.g. WT/IFNα vs. KO/INFα) using ordinary one-way analysis of variance (ANOVA) followed by Sidak’s multiple comparison test. ^**#**^, *p* < 0.05; ^**##**^, *p* < 0.01; ^**###**^, *p* < 0.001; and ^**####**^, *p* < 0.0001.

We previously established that loss of SLFN5 in GBM cells enhances ISG expression following IFN-I treatment^5^, consistent with a repressive effect of SLFN5 on IFN-I transcriptional activity. To define the requirement for different SLFN5 domains in this repressive transcriptional function, we evaluated our panel of SLFN5 deletion constructs using quantitative RT-PCR. We selected 5 ISGs (*ISG15*, *MX1*, *IRF7*, *CD274*, and *PDCD1LG2*) and monitored their mRNA expression (Fig. 2A–E). Re-expression of SLFN5WT in LN229-SLFN5KO cells restored transcriptional suppression of ISGs to levels comparable to those seen in parental LN229 cells following IFNα treatment (Fig. 2A). Re-expression of SLFN5Δ4 also suppressed ISG expression similarly (Fig. 2D). These results suggest that SLFN5Δ4 retains the ability to rescue both transcriptional repression (Fig. 2D) and GBM cell proliferation (Fig. 1D), indicating that the Walker A and B motifs are dispensable for these biological functions.

On the other hand, SLFN5Δ5, which is sequestered in the cytoplasm due to deletion of the NLS, failed to suppress ISG induction, displaying ISG expression levels similar to SLFN5KO cells after IFNα treatment (Fig. 2E). Likewise, SLFN5Δ2 lacking aa 191-304 within the Schlafen core domain including the “Schlafen box”, and SLFN5Δ3 lacking the successive aa 306–573, were both unable to restrain IFN-I induced ISG expression (Fig. 2B,C). Notably, the magnitude of derepression varied among the different ISG promoters examined (Fig. 2A–E), although the overall trends were consistent. Collectively, these results reveal a notable overlap between SLFN5 domain portions required for transcriptional repression of ISGs and those critical for promoting GBM cell proliferation. While Walker A/B motifs appear dispensable, the NLS, regions surrounding the Schlafen box (aa 191-304), and additional elements within aa 306–573 are essential for both ISG suppression and stimulation of GBM cell growth.

### Key arginine residues within the Schlafen core domain, but not the SWAVDL motif, are required for ISG repression and GBM cell proliferation

**Fig. 3:**
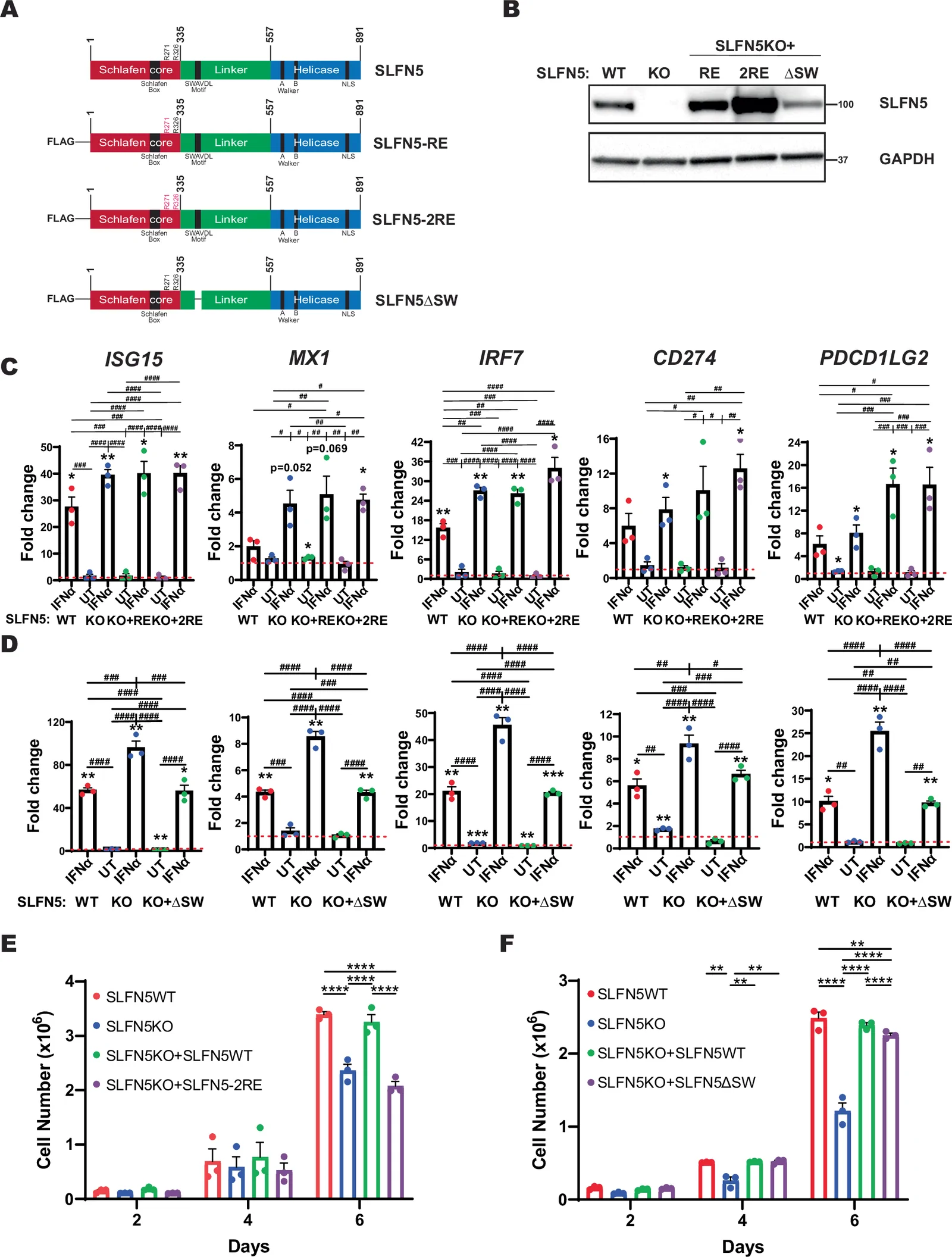
Mutational analysis of SLFN5 for ISG repression and effects on GBM proliferation. **A** Schematic of SLFN5 domain architecture and mutant constructs SLFN5R271E (RE), SLFN5R271E/R326E (2RE), and SLFN5ΔSWAVDL (SLFN5ΔSW). **B** Immunoblotting analysis using an anti-SLFN5 antibody to assess SLFN5 expression in LN229, LN229-SLFN5KO, and LN229-SLFN5KO cells stably re-expressing the indicated SLFN5 mutant constructs. Anti-GAPDH antibody was used as a loading control. LN229 (WT), LN229-SLFN5KO (KO), SLFN5KO + SLFN5RE (KO + RE), and SLFN5KO + SLFN5-2RE (KO + 2RE) (**C**) or SLFN5KO + SLFN5ΔSWAVDL (KO + ΔSW) (**D**) were serum starved for 24 h and then treated with 5000 IU/mL IFNα for 6 h. mRNA levels of *ISG15*, *MX1*, *IRF7*, *CD274*, and *PDCD1LG2* were assessed by quantitative RT-PCR using *GAPDH* for normalization. Data are expressed as relative mRNA expression of indicated genes over LN229-WT untreated cells (red dashed line) and represent means ± SEM of three independent experiments, each done in three replicates. Statistical analyses were performed using One sample *t* test to compare average fold change to FC = 1 (untreated LN229-WT). *, *p* < 0.05; **, *p* < 0.01; and ***, *p* < 0.001. In addition, statistical significance was assessed between pairs of experimental conditions (e.g. WT/IFNα vs. KO/INFα) using ordinary one-way analysis of variance (ANOVA) followed by Sidak’s multiple comparison test. ^#^, *p* < 0.05; ^##^, *p* < 0.01; ^**###**^, *p* < 0.001 and ^**####**^, *p* < 0.0001. **E** Cellular proliferation of LN229, LN229-SLFN5KO, and LN229-SLFN5KO re-expressing either SLFN5WT or SLFN5-2RE was determined by counting cells on days 2, 4, and 6 following seeding of 50,000 cells per well of a 6-well plate. Data represent means ± SEM of three independent experiments, each done in three replicates. Statistical analysis was performed using ordinary two-way analysis of variance (ANOVA) followed by Tukey’s multiple comparisons test between cell lines, and day 6 comparisons are shown. ****, *p* < 0.0001. **F** Cellular proliferation of LN229, LN229-SLFN5KO, and LN229-SLFN5KO re-expressing either SLFN5WT or SLFN5-ΔSW was determined by counting cells on days 2, 4, and 6 following seeding of 50,000 cells per well of a 6-well plate. Data represent means ± SEM of three independent experiments, each done in three replicates. Statistical analysis was performed using ordinary two-way analysis of variance (ANOVA) followed by Tukey’s multiple comparisons test. **, *p* < 0.01; ****, *p* < 0.0001.

Although SLFN5Δ2 and SLFN5Δ3 lack distinct regions of the protein, both failed to rescue the phenotypes associated with SLFN5 loss (Figs. 1D and 2B, C). The C-terminal lobe of the Schlafen core domain contains a zinc finger motif, and two adjacent arginine residues (R271 and R326) have been shown to be important for SLFN5’s nucleotide binding ability^29^. Importantly, SLFN5Δ2 and SLFN5Δ3 each lack one of these arginine residues. To test whether loss of these arginine residues may contribute to the impaired function of SLFN5Δ2 and SLFN5Δ3 deletion constructs, we generated an R271E (RE) point mutant and a double mutant R271E/R326E (2RE) (Fig. 3A). When re-expressed in LN229-SLFN5KO cells (Fig. 3B), both mutants further increased ISG expression after IFNα treatment, with levels comparable to or even exceeding those observed in SLFN5KO cells (Fig. 3C). In addition to missing R326, SLFN5Δ3 also lacks the Schlafen specific SWAVDL motif within the linker domain, potentially contributing to the failure to rescue the phenotypes observed after SLFN5 loss. To determine whether the SWAVDL motif is required for SLFN5’s transcriptional repressive function, we generated a SWAVDL deletion construct (SLFN5ΔSW) (Fig. 3A, B). In contrast to SLFN5-2RE, SLFN5ΔSW reconstituted cells were still able to restrain ISG induction after IFNα treatment, comparable to parental LN229 cells (Fig. 3D). Thus, the SWAVDL motif is dispensable for transcriptional repression. Consistent with a functional link between SLFN5-mediated transcriptional repression and cell proliferation, SLFN5-2RE also failed to restore GBM cell proliferation (Fig. 3E), while SLFN5ΔSW effectively rescued proliferative capacity (Fig. 3F). Together, these data reveal a striking functional overlap between SLFN5 elements required for transcriptional suppression and those necessary for promoting GBM cell proliferation. Thus, the loss of R271 in SLFN5Δ2 and R326 in SLFN5Δ3, rather than deletion of the SWAVDL motif, is likely responsible for the observed failure of these constructs to rescue transcriptional suppression and GBM proliferation.

### Substitution of arginine residues R271 and R326 with glutamates disrupts SLFN5 chromatin association

**Fig. 4:**
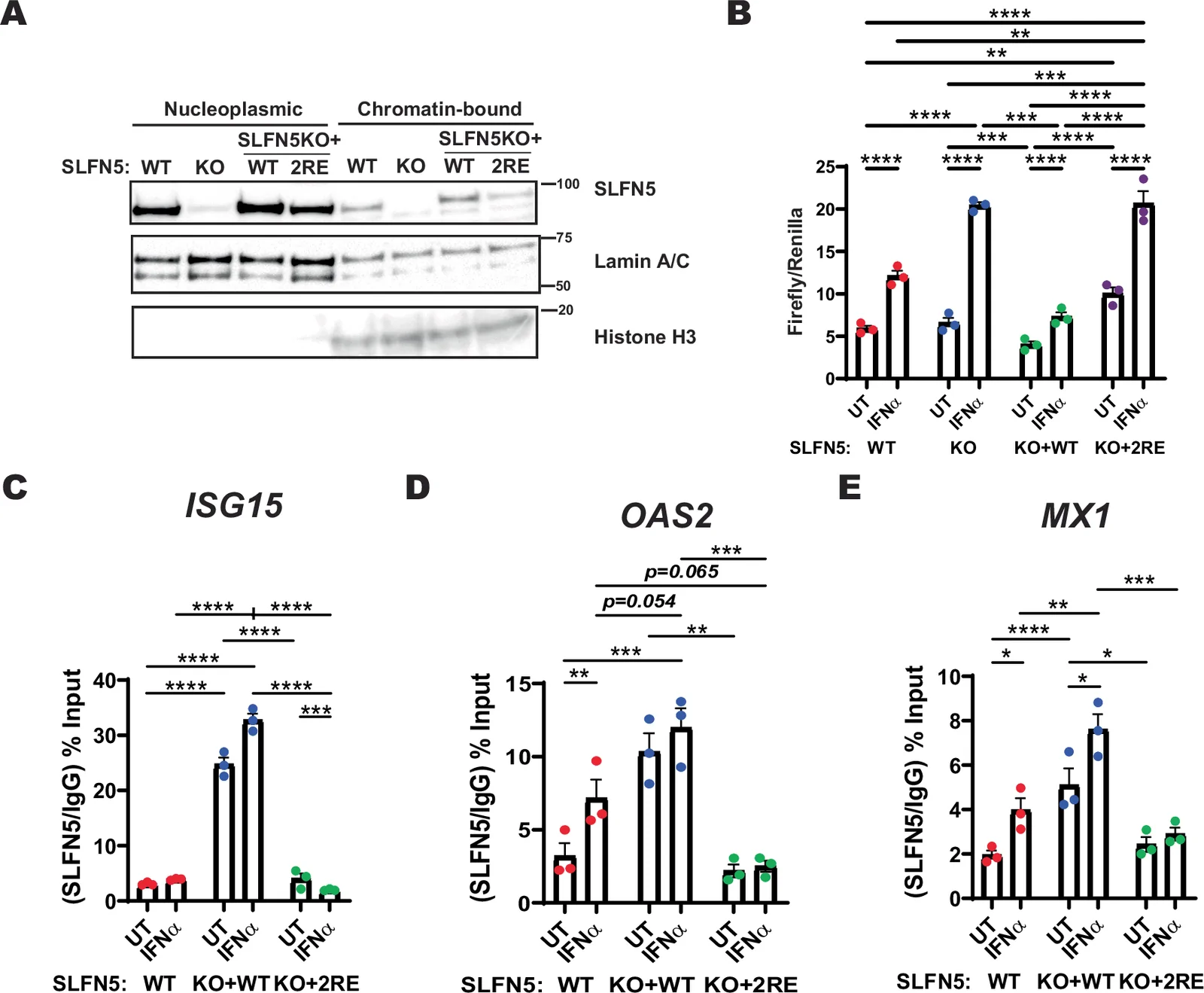
Key role for zinc finger-adjacent arginines in SLFN5 for chromatin association and repression of ISRE-driven transcription. **A** LN229, LN229-SLFN5KO, and LN229-SLFN5KO cells stably re-expressing SLFN5WT or SLFN5-2RE (2RE) were fractionated into nucleoplasmic and chromatin-bound compartments and processed for immunoblotting analysis. SLFN5 localization was assessed by immunoblotting with an anti-SLFN5 antibody. Antibodies for the nuclear marker Lamin A/C and chromatin marker histone H3 were used as controls. **B** ISRE-firefly-luciferase reporter assay using parental LN229, LN229-SLFN5KO (KO), and LN229-SLFN5KO cells stably re-expressing SLFN5WT (KO + WT) or SLFN5-2RE (KO + 2RE). Cells were untreated or treated with 5000 IU/mL IFNα for 6 hours. Data represent means ± SEM of three independent experiments, each done in three replicates. Statistical analysis was performed using two-way analysis of variance (ANOVA) followed by Tukey’s multiple comparisons test. **, *p* < 0.01; ***, *p* < 0.001; and ****, *p* < 0.0001. **C**–**E** Chromatin immunoprecipitation (ChIP) for SLFN5 in parental LN229, LN229-SLFN5KO (KO), LN229-SLFN5KO cells stably re-expressing SLFN5WT (KO + WT) or SLFN5-2RE (KO + 2RE). Cells were left untreated or treated with 5000 IU/mL IFNα for 6 hours. qPCR was performed on immunoprecipitated DNA with primers for the ISRE elements in the *ISG15* (**C**), *OAS2* (**D**), and *MX1* (**E**) promoters. Rabbit IgG antibody was used as a negative control. Data are normalized to each sample’s input DNA. Data represent means ± SEM of three independent experiments, each done in three replicates. Statistical analysis was performed using two-way analysis of variance (ANOVA) followed by Tukey’s multiple comparisons test. *, *p* < 0.05; **, *p* < 0.01; ***, *p* < 0.001; and ****, *p* < 0.0001.

Given the striking effect of the SLFN5-2RE mutant on ISG transcriptional regulation (Fig. 3C), we next investigated whether substitution of these arginine residues with glutamic acid alters SLFN5’s association with nuclear chromatin. We and others have shown that SLFN5 can bind chromatin to regulate transcription of various genes and DNA repair^5,22,31,32^. To this end, we fractioned cell lysates into nucleoplasmic and chromatin-bound compartments. In the nucleoplasmic fraction, comparable amounts of endogenous SLFN5 and re-expressed constructs were detected. Remarkably, chromatin-bound SLFN5-2RE was markedly reduced as compared to both endogenous and reconstituted SLFN5WT (Fig. 4A). These results strongly suggest that the SLFN5-2RE mutant exhibits reduced affinity for nuclear chromatin, impairing its ability to efficiently repress ISG transcriptional responses.

### SLFN5 represses IFN-I-driven transcription through interferon-sensitive response element (ISRE) promoter binding

Given that most ISG promoters contain interferon-sensitive response elements (ISRE), we employed a firefly-luciferase reporter driven by an ISRE-containing promoter, along with a Renilla-luciferase construct for normalization, to assess ISRE-dependent transcriptional activity. Consistent with our previous results (Fig. 3C), both SLFN5KO cells as well as SLFN5-2RE reconstituted cells failed to suppress IFN-I-induced firefly-luciferase (Fig. 4B). To directly assess SLFN5 binding to ISRE-containing promoters, we performed chromatin immunoprecipitation (ChIP) experiments using primers for ISRE sites within *ISG15* (Fig. 4C), *OAS2* (Fig. 4D), and *MX1* (Fig. 4E) promoters. ChIP analysis revealed that SLFN5 occupancy at these promoters was markedly reduced in cells re-expressing the SLFN5-2RE mutant as compared to cells reconstituted with SLFN5WT (Fig. 4C–E), with some variability across the promoters tested. This variability could be due to the fact that SLFN5 promoter occupancy was assessed in a reconstitution system and at selected loci, and therefore may not fully capture the dynamic, cofactor-dependent, and time-dependent chromatin interactions that may be involved in SLFN5-mediated ISG regulation. Nevertheless, these results indicate that SLFN5 represses IFN-I-driven transcriptional responses through chromatin association at ISRE-containing promoters. Importantly, our results provide compelling evidence for the two arginine residues adjacent to the zinc finger motif being critical for SLFN5’s ability to bind chromatin and for its transcriptional repressor function. Further, these two arginine residues are also crucial for SLFN5 to stimulate GBM cell proliferation.

### Cross-species functional conservation of SLFN5-mediated transcriptional repression and cancer cell proliferation in GBM

**Fig. 5:**
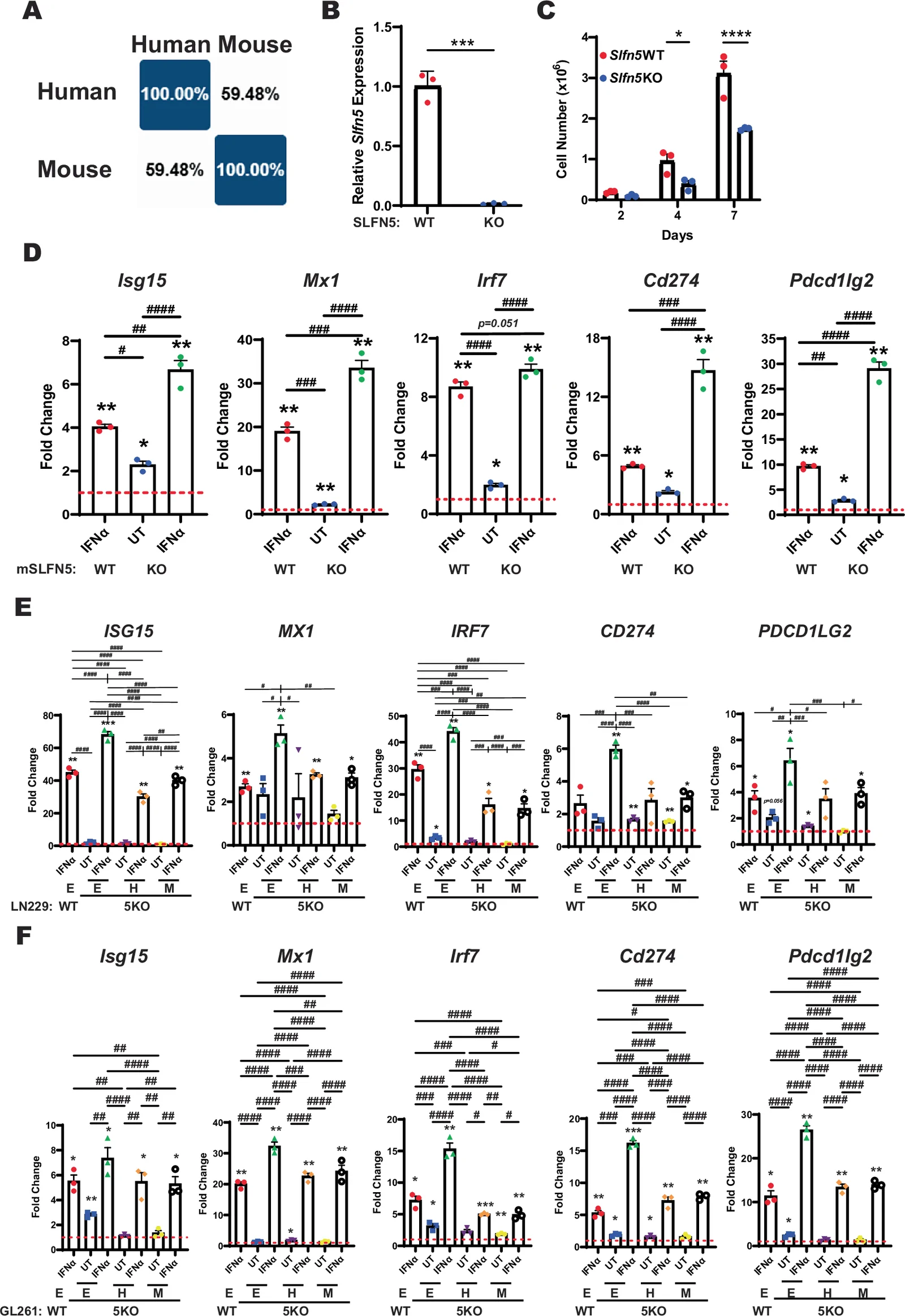
Mouse SLFN5 restricts interferon signaling in both mouse and human glioblastoma cells. **A** Protein Percent Identity Matrix between human (UniProtKB: Q08AF3) and mouse (UniProtKB: Q8CBA2) SLFN5 using the UniProtKB database Align tool (https://www.uniprot.org/align). **B** *Slfn5* mRNA expression of GL261 WT (WT) and GL261-SLFN5KO (KO) cells was assessed by quantitative RT-PCR using *Gapdh* for normalization. Data are expressed as relative mRNA expression of *Slfn5* and represent means ± SEM of three independent experiments, each done in three replicates. Statistical analysis was performed using unpaired *t*-test. ***, *p* < 0.001. **C** GL261 and GL261-SLFN5KO cells were seeded in 6-well plates (50,000 cells per well) followed by cell counting on days 2, 4, and 7 to assess mouse GBM cellular proliferation. Data represent means ± SEM of three independent experiments, each done in three replicates. Statistical analysis was performed using ordinary two-way analysis of variance (ANOVA) followed by Sidak’s multiple comparisons test and comparisons for day 7 are shown. *, *p* < 0.05; ****, *p* < 0.0001. **D** GL261 (WT) and GL261-SLFN5KO (KO) cells were serum starved for 24 hours and then treated with 5000 IU/mL murine IFNα for 6 hours. mRNA expression of *Isg15*, *Mx1*, *Irf7*, *Cd274*, and *Pdcd1lg2* was assessed by quantitative RT-PCR using *Gapdh* for normalization. Data are expressed as relative mRNA expression of indicated genes over GL261-WT untreated cells (red dashed line) and represent means ± SEM of three independent experiments, each done in three replicates. Statistical analyses were performed using One sample *t* test to compare average fold change to FC = 1 (untreated GL261-WT). *, *p* < 0.05; **, *p* < 0.01. In addition, statistical significance was assessed between pairs of experimental conditions (e.g. WT/IFNα vs. KO/INFα) using ordinary one-way analysis of variance (ANOVA) followed by Tukey’s multiple comparison test. ^**#**^, *p* < 0.05; ^**##**^, *p* < 0.01; ^**###**^, *p* < 0.001; and ^**####**^, *p* < 0.0001. **E** LN229 (WT) and LN229-SLFN5KO (5KO) cells were transiently transfected with either 3XFLAG-EGFP (E), 3XFLAG-hSLFN5 (H), or 3XFLAG-mSLFN5 (M) and serum starved for 24 h and then treated with 5000 IU/mL human IFNα for 6 hours. mRNA expression of *ISG15*, *MX1*, *IRF7*, *CD274*, and *PDCD1LG2* was assessed by quantitative RT-PCR using *GAPDH* for normalization. Data are expressed as relative mRNA expression of indicated genes over LN229-WT-3XFLAG-EGFP untreated cells (red dashed line) and represent means ± SEM of three independent experiments, each done in three replicates. Statistical analyses were performed using One sample *t* test to compare average fold change to FC = 1 (untreated LN229-WT-3XFLAG-EGFP). *, *p* < 0.05; **, *p* < 0.01; and ***, *p* < 0.001. In addition, statistical significance was assessed between pairs of experimental conditions (e.g. WT/IFNα vs. KO/INFα) using ordinary one-way analysis of variance (ANOVA) followed by Tukey’s multiple comparison test. ^**#**^, *p* < 0.05; ^**##**^, *p* < 0.01; ^**###**^, *p* < 0.001; and ^**####**^, *p* < 0.0001. **F** GL261 (WT) and GL261-SLFN5KO (5KO) cells were transiently transfected with either 3XFLAG-EGFP (E), 3XFLAG-hSLFN5 (H), or 3XFLAG-mSLFN5 (M) and serum starved for 24 h and then treated with 5,000 IU/mL murine IFNα for 6 h. mRNA expression of *Isg15*, *Mx1*, *Irf7*, *Cd274*, and *Pdcd1lg2* was assessed by quantitative RT-PCR using *Gapdh* for normalization. Data are expressed as relative mRNA expression of indicated genes over GL261-WT-3XFLAG-EGFP untreated cells (red dashed line) and represent means ± SEM of three independent experiments, each done in three replicates. Statistical analyses were performed using One sample *t* test to compare average fold change to FC = 1 (untreated GL261-WT-3XFLAG-EGFP). *, *p* < 0.05; **, *p* < 0.01; and ***, *p* < 0.001. In addition, statistical significance was assessed between pairs of experimental conditions (e.g. WT/IFNα vs. KO/INFα) using ordinary one-way analysis of variance (ANOVA) followed by Tukey’s multiple comparison test. ^**#**^, *p* < 0.05; ^**##**^, *p* < 0.01; ^**###**^, *p* < 0.001; and ^**####**^, *p* < 0.0001.

To date, six human *SLFN* genes and nine mouse *Slfn* genes have been identified^12,17^. Of these, only *SLFN5* and *SLFN14* are found in both species. Amino acid sequence comparison indicated that mouse SLFN5 protein is approximately 59.48% identical to human SLFN5 (Fig. 5A and Supplementary Fig. 1). However, direct experimental evidence to define whether these proteins may act as functional orthologs is still lacking. To better understand whether mSLFN5 may represent the functional ortholog of hSLFN5 in the context of IFN-I signaling, we assessed its capability to exert similar transcriptional repressive effects. We subjected the mouse GBM cell line GL261 to CRISPR-Cas9 gene editing to generate mSLFN5 knockout cells (GL261-SLFN5KO) (Fig. 5B). Similar to our observation in human GBM cells (Fig. 1D) loss of mSLFN5 in GL261 cells resulted in a significantly slower proliferation rate compared to parental GL261 cells (Fig. 5C). Also, mSLFN5-deficient GL261 cells had increased expression of murine ISGs (*Isg15, Mx1, Irf7, Cd274*, and *Pdcd1lg2*) following mouse IFNα treatment (Fig. 5D). Remarkably, re-expression of mSLFN5 in human LN229-SLFN5KO cells (Supplementary Fig. 2A) suppressed transcription of ISGs comparable to parental LN229 and cells re-expressing hSLFN5 (Fig. 5E). Conversely, in the reciprocal experiment, re-expression of hSLFN5 in murine GL261-SLFN5KO cells (Supplementary Fig. 2B) restored ISG repression (Fig. 5F). These results provide strong evidence for mouse SLFN5 representing the functional ortholog of human SLFN5 in the context of modulating cancer cell proliferation and IFN transcriptional responses in GBM.

### Structural comparison of human and mouse SLFN5 proteins

SLFN5 comprises an N-terminal Schlafen core domain containing the Schlafen box, followed by a linker domain with the SWAVDL motif, and a C-terminal helicase domain with Walker A/B motifs and an NLS (Supplementary Fig. 3A). To assess structural conservation between species, we performed pairwise structural superposition of human and mouse SLFN5 fold models using Matchmaker in UCSF ChimeraX (Supplementary Fig. 3B). After pruning of poorly aligned atom pairs, superposition of 745 matched atom pairs resulted in a root-mean-square deviation (RMSD) of 0.934 Å, consistent with high degree of structural similarity between human and mouse SLFN5. Additionally, the structural models of human and mouse SLFN5 were predicted using AlphaFold3 with a confidence score (pLDDT) of 86.7 and 84.3, respectively. Together with amino acid sequence alignment (Fig. 5A and Supplementary Fig. 1), this three-dimensional structural comparison further corroborates a conserved overall architecture of human and mouse SLFN5 (Supplementary Fig. 3B). In human SLFN5, the two positively charged arginine residues, R271 and R326, are surface exposed, consistent with a role in accommodating chromatin (Supplementary Fig. 3C). In mouse SLFN5, these residues are replaced by neutral asparagine residues (Supplementary Figs 1 and 3D). Substitution with glutamic acids in the SLFN5-2RE mutant introduces locally exposed negative charge (Supplementary Fig. 3E), which would be expected to repel the negatively charged DNA backbone, providing a mechanistic explanation for the failure of the SLFN5-2RE mutant to restore transcriptional repression.

## Discussion

SLFN5 has attracted increasing attention due to its diverse pathophysiological roles in human malignancies^12^. Our previous work demonstrated that SLFN5 promotes tumorigenesis in PDAC through E2F7-mediated transcriptional regulation^23^ and contributes to GBM malignancy, in part by repressing IFN-I transcriptional responses^5^. The role of SLFN5 as a transcriptional regulator has been corroborated by additional studies showing its ability to suppress transcription of viral pathogens such HIV-1^20^ and herpes simplex virus type 1 (HSV-1)^30^, as well as eukaryotic transcription factors including ZEB1^22^ and activating transcription factor 4 (ATF4)^31^. Still, the specific SLFN5 domains and structural motifs required for SLFN5’s transcriptional regulatory activity remain poorly defined.

Subgroup III SLFNs, such as SLFN5 and SLFN11, share a conserved C-terminal helicase domain including Walker A/B motifs with ATPase activity. Mutations in the canonical Walker A and B motifs of SLFN11 (K605M/D668A or E669Q) abrogate key functions such as chemosensitivity to DNA-damaging agents (DDAs) and replication fork degradation^15,33,34^. Similarly, mutation within the Walker B motif of SLFN5 (D649A) disrupts its ability to facilitate non-homologous end joining (NHEJ)^32^. Here we show that the SLFN5Δ4 construct, lacking the C-terminal ATPase domain, retains the ability to repress ISG transcription and to promote GBM cell proliferation. This is in line with the observation that the C-terminal ATPase domain of SLFN5 is also dispensable for inhibition of viral production^20^. These findings support a model in which the C-terminal helicase activity of SLFN5 may be essential for the remodeling of RNA/DNA strands but is not required for its transcriptional regulatory function and for cancer cell proliferation.

Most human SLFN proteins exhibit two catalytically active domains: a C-terminal helicase domain and an N-terminal tRNA/rRNA endoribonuclease activity^25,26^. This ribonuclease activity enables SLFN11 to suppress type II tRNAs^25^, SLFN13 to cleave tRNAs^26^, SLFN12 to target rRNAs^27^, and SLFN14 to degrade tRNAs, rRNAs, and ribosome-associated mRNAs^28^. SLFN5 is unique because structural analyses suggested it lacks the N-terminal endoribonuclease activity^26^, and subsequent studies confirmed that the SLFN5 core domain neither binds nor hydrolyzes ATP^29^. In the current study, we investigated the functional role of the SLFN5 core domain and found strong evidence that it contributes to transcriptional regulation. Using a series of SLFN5 deletion constructs that were re-expressed in SLFN5KO cells, we observed that deletions within the N-terminal Schlafen core domain led to increased ISG expression following IFNα treatment. The cryo-EM structure of SLFN5 provided evidence that two arginine residues (R271 and R326) near the zinc finger motif are critical for SLFN5 DNA affinity^29^. In line with this notion, we found that substitution of these positively charged arginine residues with negatively charged glutamates (SLFN5-2RE) exhibited greatly reduced association with nuclear chromatin and with ISRE-containing promoters. Remarkably, SLFN5-2RE phenocopied the deletion mutants SLFN5Δ2 and SLFN5Δ3 in terms of ISG expression and GBM cell proliferation, indicating that an intact N-terminal Schlafen core domain is required for transcriptional repressive effects. Interestingly, a previous observation by another group had shown that a truncated nuclear SLFN5 construct (SLFN5-1-355 + NLS) encompassing the entire N-terminal Schlafen core domain still retained the ability to repress HIV-1 transcription^20^. Collectively, these results may explain why the N-terminal endoribonuclease activity present in other subgroup III SLFNs is dispensable for SLFN5’s key biological functions. Instead, they highlight the critical role of the catalytically inactive SLFN5 core domain, particularly residues near the zinc finger motif, in mediating DNA binding, enrichment on ISRE-containing promoters, transcriptional modulation, and cancer cell proliferation, while the conserved SWAVDL motif and C-terminal helicase activity appear dispensable for these functions.

Our findings also extend the concept of SLFN5 as a negative regulator of immune signaling because loss of SLFN5 led to upregulation of a plethora of canonical ISGs, including the immune checkpoint genes *CD274* (PD-L1) and *PDCD1LG2* (PD-L2) (Fig. 2)^5^. Elevated expression of PD-L1 and PD-L2 is often predictive of responses to immune checkpoint inhibitors (ICIs)^35–39^. Additionally, ICI effectiveness has been shown to depend on intact IFN-I signaling, as evidenced by impaired response to combinatorial cytotoxic T-lymphocyte-associated protein 4 (CTLA-4) and PD-L1 blockade in mouse models with impaired IFN-I responses^40^. We observed that loss of SLFN5 in GBM cells results in increased basal ISG expression even in the absence of exogenous IFNα stimulation, indicating a constitutive repressive role of SLFN5 on these genes. Together, these findings further support our notion of SLFN5 potentially acting as an “intracellular immune checkpoint” through its ability to restrain IFN downstream signaling^12,24^.

Translational in vivo studies of SLFNs are complicated by interspecies variability within the SLFN gene family. Currently, humans encode six *SLFN* genes, whereas mice encode nine^12^, with several members lacking direct orthologs. For instance, *SLFN11* is unique to humans and plays key roles in chemotherapy response and replication stress sensing^15,41^, while *Slfn2* is specific for mice and regulates T cell development^42^. Although mouse *Slfn8* and *Slfn9* can partially compensate for *SLFN11* loss in the context of replication stress^43^, they are phylogenetically closer to human *SLFN13* than to *SLFN11*^44,45^, highlighting the complexity of functional conservation across species within the SLFN family. Here, structural modeling revealed a conserved architecture between human and murine SLFN5, and the low RMSD of 0.934 Å over 745 pruned atom pairs indicates that the backbone architecture of SLFN5 is highly conserved between human and mouse. In line with this, our biological analyses provide additional strong evidence that mouse SLFN5 serves as the functional ortholog of human SLFN5 in the context of IFN-I signaling. Genetic disruption of mSLFN5 leads to enhanced ISG expression, mirroring the phenotype observed with hSLFN5 loss. Furthermore, re-expression of mSLFN5 in human LN229-SLFN5KO cells, or reciprocally hSLFN5 in murine GL261-SLFN5KO cells, restored ISG repression, strongly indicating functional conservation. These findings establish the utility of syngeneic murine models for future exploration of SLFN5 and its therapeutic potential in immune regulation and cancer.

## Methods

### Cell lines and reagents

The human glioblastoma cell line LN229 was obtained from ATCC, and the murine glioblastoma cell line GL261 was a gift from Dr. Maciej S. Lesniak (Northwestern University, Chicago, IL), who obtained it from NCI Frederick National Tumor Repository Laboratory. The Lenti-X 293T cell line was obtained from Clontech, and all cell lines were grown in DMEM supplemented with 10% fetal bovine serum (FBS) and gentamicin. All cell lines were grown at 37 °C and 5% CO_2_. Cells were tested every 6 months by short-tandem repeat (STR) analysis and authenticated according to published STR profiles. Cell lines were regularly tested for *Mycoplasma*. A detailed list of reagents can be found in Supplementary Table 1.

### RNA isolation and real-time quantitative PCR

Total RNA was isolated using RNeasy Mini Kit (QIAGEN); 2 µg of total RNA was reverse transcribed into cDNA using the High Capacity cDNA Reverse Transcription Kit (Applied Biosystems). A list of Taqman Probes (ThermoFisher) can be found in Supplementary Table 1. *GAPDH* was used as a normalization control. Quantitative PCR reactions were performed on a QuantStudio 6 Flex real-time PCR system (ThermoFisher) with the following condition: activation at 50 °C; 2 min and 95 °C; 20 s, 50 cycles of denaturation at 95 °C; 1 s and annealing/extension at 60 °C; 20 s. TaqMan Fast Advanced Master mix (ThermoFisher) was used to carry-out quantitative PCR reactions. Relative gene expression was analyzed by the ^ΔΔ^C_t_ method.

### GBM cell proliferation assay

Cells were seeded at 50,000 cells per 6-well. At 2, 4, and 6 days after seeding, cells were enzymatically dissociated, stained with trypan blue, and counted on a hemacytometer. For each time point, 3 wells were analyzed.

### Cell lysis and immunoblotting

Cells were lysed in lysis buffer (50 mM Tris, pH 7.5, 150 mM NaCl, 5 mM EDTA, 0.5% NP40), supplemented with protease (EMD Millipore Protease Cocktail III) and phosphatase (10 mM sodium fluoride, 1 mM sodium orthovanadate) inhibitors. Lysates were centrifuged (15,000 rpm, 20 min, 4 °C) and protein concentrations were measured with Bio-Rad Protein Assay. Equal amounts of protein were mixed with SDS buffer and denatured by heating prior to resolving by SDS-PAGE. Proteins were transferred to PVDF membranes, blocked with 5% nonfat milk at room temperature for 1 hour, incubated with primary antibodies (anti-SLFN5, 1:500; anti-Flag-M2-HRP, 1:2,000; anti-GAPDH, 1:20,000) overnight in 5% bovine serum albumin (BSA) at 4 °C, and probed with HRP-conjugated secondary antibodies (Goat anti-Mouse IgG(H + L)-HRP, 1:10,000; anti-Rabbit IgG-HRP, 1:2,000) for 1 h at room temperature. Chemiluminescence was detected using a ChemiDoc MP imager (Bio-Rad). A list of antibodies used can be found in Supplementary Table 1.

### Generation of SLFN5KO and Slfn5KO cells using CRISPR/Cas9 technology

LN229 (human) and GL261 (mouse) cells were transfected using Turbofect (ThermoFisher) with plasmids containing Cas9, single-guide RNA (sgRNA) targeting human *SLFN5 or* mouse *Slfn5*, and a homology direct repair containing a Puromycin resistance gene and red fluorescent protein (RFP) (For LN229 *SLFN5* CRISPR/Cas9 KO plasmid (h2, sc-408333-KO-2) and *SLFN5* HDR plasmid (h2, sc-408333-HDR-2); For GL261 *Slfn5* CRISPR/Cas9 KO plasmid (sc-435875) and *Slfn5* HDR plasmid (sc-435875-HDR)). These plasmids were purchased from Santa Cruz Biotechnology. Cells were kept under Puromycin (2 μg/mL) selection for 2 weeks and then sorted for expression of red fluorescent protein using fluorescent activated cell sorting (FACS).

### Generation of FLAG-SLFN5 constructs

pReciever-M2-3XFLAG-*SLFN5* (GeneCopoeia) was used as a PCR template to flank the *SLFN5* ORF with restriction sites BamHI and KpnI (New England Biolabs) for cloning into the pENTR4-FLAG (w210-2) vector (gift from Eric Campeau & Paul Kaufman, Addgene)^46^. FLAG-*SLFN5* was further cloned into pLenti-CMV-Hygro-DEST (w117-1) (gift from Eric Campeau & Paul Kaufman, Addgene) using Gateway cloning^46^. FLAG-*SLFN5* deletion and point mutation constructs were generated using the Q5® Site-Directed Mutagenesis Kit (New England Biolabs) and pENTR4-FLAG-*SLFN5* plasmid. Primers were generated using NEBaseChanger (https://nebasechanger.neb.com). For a list of primers, see Supplementary Table 1.

### Generation of LN229 cells stably re-expressing FLAG-SLFN5 constructs

Lentiviruses were generated using Lenti-X Packaging Single Shots (VSV-G) (Clontech) and Lenti-X 293T (293T) cells according to the manufacturer’s instruction. Briefly, 5.0 × 10^6^ 293T cells were seeded in a 10 cm dish containing 8 mL complete media. The next day, 7 μg lentiviral plasmid was diluted with sterile nuclease-free water to a final volume of 600 μL, added to a Lenti-X Packaging Single Shot vial, vortexed and incubated for 10 minutes at room temperature. Mixture was added dropwise to 293T cells and left overnight at 37 °C. On day 3, 5 mL complete media was added to the 10 cm dish and left for an additional 3 days in the incubator. On day 6, the lentiviral titer was estimated using Lenti-X GoStick (Clontech). 20 μL of media containing lentivirus particles was added to a GoStick cassette, followed by 80 μL Chase Buffer 1, and allowed to flow through the cassette for 10 min. To concentrate lentiviral particles, media was filtered through a 0.45 μm polyethersulfone filter into a 50 mL conical tube and combined with 1/3 volume of Lenti-X Concentrator (Clontech) and incubated overnight at 4 °C. The following day, the concentrated lentivirus was centrifuged at 1,500 × g for 1 h at 4 °C. Lentivirus pellet was resuspended at 1/50 of the original volume with DMEM and stored at −80 °C. LN229-SLFN5KO cells were transduced with lentiviruses carrying FLAG-SLFN5-Hygro deletion or point mutation using TransDux Max Lentivirus Transduction enhancer (System Biosciences) according to manufacturer’s protocol. Cells were selected with 2 μg/mL Puromycin and 200 μg/mL Hygromycin for 10 days. Expression of FLAG-SLFN5 was verified by immunoblotting with a SLFN5 antibody (anti-SLFN5, 1:500).

### Subcellular fractionation

Equal volume cell pellets were fractionated into either cytoplasmic and nuclear fraction using the NE-PER^TM^ Nuclear and Cytoplasmic Extraction Reagent Kit (ThermoFisher catalog #78833) or nucleoplasmic and chromatin-bound fractions according to the Subcellular Protein Fractionation Kit for Cultured Cells (ThermoFisher catalog #78840). Pellets between fractions were rinsed with 1X PBS. Purity of fractions was assessed using HSP90 for cytoplasmic proteins (anti-HSP90, 1:1,000), Lamin A/C for nuclear proteins (anti-Lamin A/C, 1:1,000), and Histone H3 for chromatin-bound fraction (anti-Histone H3, 1:1,000).

### Confocal laser scanning microscopy

Cells were seeded onto 0.17 mm coverslips (Electron Microscopy Sciences) in 12-well plates (30,000 cells/well). After 48 hours, coverslips were fixed in 4% paraformaldehyde (PFA), permeabilized with 1% Triton-X 100 in phosphate-buffered saline (PBS) for 15 min and blocked in a solution of 1% BSA in PBS, followed by incubation with rabbit anti-SLFN5 antibody (Sigma-Aldrich, 1:200) for 1.5 h at room temperature. Subsequently, coverslips were incubated with secondary goat anti-rabbit-AF488 antibody (ThermoFisher, 1:2,000) for 1 hour. Stained coverslips were mounted with ProLong® Diamond Antifade Mountant with 4’,6-diamidino-2-phenylindole (DAPI) (Life Technologies). Images were acquired on a Nikon A1plus inverted microscope equipped with a Plan Apo 40× oil objective lens from Nikon with NA 1.3. For microscopic analysis, the acquisition software NIS-Elements was used.

### Luciferase reporter assays

LN229, LN229-5KO, LN229-5KO cells stably re-expressing SLFN5WT, or SLFN5-2RE were co-transfected with pGF1-ISRE-luciferase and pRL-TK Renilla constructs (at a ratio of 10:1) using TurboFect transfection reagent according to the manufacturer’s instructions. Forty-eight hours after transfection, triplicate cultures were left untreated or treated with human IFNα (5000 IU/ml) for 6 h. The cells were lysed, and luciferase and Renilla activity were measured using the Dual-Luciferase® Reporter Assay System (Promega) per the manufacturer’s instructions. Luciferase activity was normalized by Renilla activity.

### *Chromatin* immunoprecipitation *(ChIP)*

ChIP was performed using the SimpleChIP Enzymatic Chromatin IP Kit with Magnetic Beads (Cell Signaling Technology), as per the manufacturer’s instructions. Formaldehyde was used to cross-link protein to DNA, glycine was added to stop the reaction. Cells were suspended in ChIP lysis buffer, and cell lysate was sonicated to generate DNA fragments and then immunoprecipitated with antibody for human SLFN5 (Sigma-Aldrich, 1 μg) or normal rabbit IgG (Cell Signaling, 1 μg). qPCR was performed on purified immunoprecipitated DNA for the *ISG15, OAS2*, and *MX1* promoters using PowerTrack™ SYBR™ Green Master Mix (ThermoFisher) according to the manufacturer’s instructions. All qPCR signals were normalized to the input DNA. For a list of primers, see Supplementary Table 1.

### Computational model building

Sequences for human (AF-Q08AF3-F1-v6) and mouse (AF-Q8CBA2-F1-v6) SLFN5 were obtained from the AlphaFold Protein Structure database (https://alphafold.ebi.ac.uk/). Modeling of wild-type, R271/R326 mutant human SLFN5, and wild-type mouse SLFN5 was performed using AlphaFold3^47^. Five predictions were generated, and the prediction with the highest pLDDT (predicted local distance difference test) was used for structural analysis. pLDDT scores ≥ 90 were considered very high confidence, while pLDDT scores < 50 were considered to have very low confidence. Structural illustrations and analyses were prepared using UCSF ChimeraX 1.11^48^.

### Statistics and reproducibility

All statistical analyses were performed using GraphPad Prism v9.0. Sample sizes, number of replicates and the appropriate statistical tests used are described in detail in each figure legend.

### Ethics

This study was designed to address biological and technical questions using in vitro models. No human participants or animals were directly involved, and no demographic, race, ethnicity, or other socially defined variables were collected or analyzed.

### Reporting summary

Further information on research design is available in the Nature Portfolio Reporting Summary linked to this article.

## Supplementary information

Supplementary Information

Description of Additional Supplementary files

Supplementary Data

Reporting Summary

## Data Availability

The data that support the findings of this study are available in the main text or the supplemental materials; values for all data points in graphs are reported in the Supplementary Data.
