# Supplementary material for: SLFN5 restrains type I interferon responses and promotes glioblastoma via its N-terminal Schlafen core domain
Source: Commun Biol. 2026 May 27;9:1128. doi: 10.1038/s42003-026-10378-7 (PMC13493812; doi:10.1038/s42003-026-10378-7)
Supplement: Supplementary file 1 [file 42003_2026_10378_MOESM1_ESM.pdf]

Supplementary Figure 1

```
SLFN5_HUMAN      MSLRIDVDTNFPECVVDAGKVTLGTQQRQEMDPRLREKQNEIILRAVCALLNSGGGIIKA      60
SLFN5_MOUSE      MSFLEDLELNFAECIQDGGKATLGVRQREEMDTTHCMKQNEIISQAVCALLNSGGGVVRV      60
                 **:  *:: ** **: *.**.***.:**:***     ****** :***********:::.

SLFN5_HUMAN      EIENKGYNYERHGVGLDVPPIFRSHLDKMQKENHFLIFVKSWNTEA-GVPLATLCSNLYH      119
SLFN5_MOUSE      EIENGDYNFERDGVGLNLPPLFRNHLDEMLYGKLFLIYVSSWDVAASHVRLATLCSNLYH      120
                 **** .**:**.****::**:**.***:*   : ***:*.**:. *  * **********

SLFN5_HUMAN      RERTSTDVMDSQEALAFLKCRTQTPTNINVSNSLGPQAAQGSVQYEGNINVSAAALFDRK      179
SLFN5_MOUSE      RCGTFTEVMDPEKALKFLKRV-QDPRILGDSDSLNLQEA----PVDDAQMILASDLFHSP      175
                 *  * *:*** ::** ***   * *  :. *:**. * *      :.   : *: **.

                                                     Schlafen box
SLFN5_HUMAN      RLQYLEKLNLPESTHVEFVMFSTDVSHCVKDRLPKCVSAFANTEGGYVFFGVHDETCQVI      239
SLFN5_MOUSE      QLQYLEKLNFTKSSHVEFQMFSADLSQGIRERLPKCVSALANSEGGYVFFGVHDETRHVI      235
                 :********: :*:**** ***:*:*: :::********:**:************* :**

                                                Zinc
                                               finger   R271 hSLFN5
SLFN5_HUMAN      GCEKEKIDLTSLRASIDGCIKKLPVHHFCTQRPEIKYVLNFLEVHDKGALRGYVCAIKVE      299
SLFN5_MOUSE      GCEKEKINCTNLKSTIDACIRKMPVYHFCGQNHKVQYELKFLEVYDKEALHGYVCAIKVE      295
                 *******: *.*:::**.**:*:**:*** *. :::* *:****:** **:*********

                   Zinc
                  finger                R326 hSLFN5
SLFN5_HUMAN      KFCCAVFAKVPSSWQVKDNRVRQLPTREWTAWMMEADPDLSRCPEMVLQLSLSSATPRSK      359
SLFN5_MOUSE      RFCCAAFAKAPDSWEIKDNNKKPLTANDWASRMIEINPDLSSFPQMIPWKSMLNTTPCSK      355
                 :****.***.*.**::***. : * :.:*:: *:* :****  *:*:   *: .:** **

SLFN5_HUMAN      PVCIHKNSECLKEQQKRYFPVFSDRVVYTPESLYKELFSQHKGLRDLINTEMRPFSQGIL      419
SLFN5_MOUSE      TVFTHKYLKCVEDLQKDYFPVSPNRITYTPESVYKDLFADYRGLRNLINMEMRCFSQGIL      415
                  *  **  :*::: ** ****  :*:.*****:**:**:::***:*** *** ******

                     SWAVDL
                      motif
SLFN5_HUMAN      IFSQSWAVDLGLQEKQGVICDALLISQNNTPILYTIFSKWDAGCKGYSMIVAYSLKQKLV      479
SLFN5_MOUSE      IFSHSWAVDLGLQRRQDVICDALLISPNNVPILYTICNKWDLGNRHYSMKVARTLKQKLV      475
                 ***:*********.:*.********* **.****** .*** * : *** ** :******

SLFN5_HUMAN      NKGGYTGRLCITPLVCVLNSDRKAQSVYSSYLQIYPESYNFMTPQHMEALLQSLVIVLLG      539
SLFN5_MOUSE      NMGGYPGRLGIIPLVLPLGSHQRVRN--DLEMPVYPESYNFITTQQVEALLQSLVIILFG      533
                 * *** *** * ***  *.*.::.:.  .  : :*******:* *::*********:*:*

                                                          Walker A
SLFN5_HUMAN      FKSFLSEELG--SEVLNLLTNKQYELLSKNLRKTRELFVHGLPGSGKTILALRIMEKIRN      597
SLFN5_MOUSE      FRPLLNEELNLESETVALLSDQQYGLLSTNLSKHREMFVHGLPGSGKTTLALMIVGKIRN      593
                 *: :*.***.  **.: **:::** ***.** * **:*********** *** *: ****

                                                                   Walker B
SLFN5_HUMAN      VFHCEPANILYICENQPLKKLVSFSKKNICQPVTRKTFMKNNFEHIQHIIIDDAQNFRTE      657
SLFN5_MOUSE      VFNCQADNILYICENQSLKRFI--VRKNVCQAVTRKTFMKNTFDNVQHIIVDEAQNFRTE      651
                 **:*:  ********* **:::   :**:** *********.*:::****:*:*******

SLFN5_HUMAN      DGDWYGKAKFITQTARDGPGVLWIFLDYFQTYHLSCSGLPPPSDQYPREEINRVVRNAGP      717
SLFN5_MOUSE      DGNWYAKAKAITQRARDGPGVLYIFLDYFQINHLCCSGLPELQHQKPLLKLTRMLRSGDN      711
                 **:**.*** *** ********:*******  **.*****  ..* *  ::.*::*...

SLFN5_HUMAN      IANYLQQVMQEARQNPPPNLPPGSLVMLYEPKWAQGVPGNLEIIEDLNLEEILIYVANKC      777
SLFN5_MOUSE      ITSYLQDIMHQIRENPPPNVPQEALMVGEELEWGPDVTGNLEITDNLNLEQMSVYVAEKC      771
                 *:.***::*:: *:*****:*  :*::  * :*. .* ***** ::****:: :***:**

                                                      NLS
SLFN5_HUMAN      RFLLRNGYSPKDIAVLFTKASEVEKYKDRLLTAMRKRKLSQLHEESDLLLQIGDASDVLT      837
SLFN5_MOUSE      QSLWRSGYFT-DVAVLFTRARDIEKCRDKLLLAMRRRSMSQLAEEPSLLVQVREGLDSLG      830
                 : * *.**   *:*****:* ::** :*:** ***:*.:*** ** .**:*: :. * *

SLFN5_HUMAN      DHIVLDSVCRFSGLERNIVFGINPGVAPPAGAYNLLLCLASRAKRHLYILKASV    891
SLFN5_MOUSE      SHVVLESVHRFSGMERSIVFGIIPMGSETAIFYNALLCLASRARTHLYIVKVVF    884
                 .*:**:** ****:**.***** *  :  *  ** ********: ****:*. .
```

Supplementary Figure 1. Sequence alignment of human and mouse SLFN5. The Schlafen box is indicated in red, the zinc finger residues are indicated in blue, the SWAVDL motif in green, the Walker A/B motifs in yellow and the nuclear localization signal (NLS) in orange. The human SLFN5 residues R271 and R326 are indicated.

Supplementary Figure 2

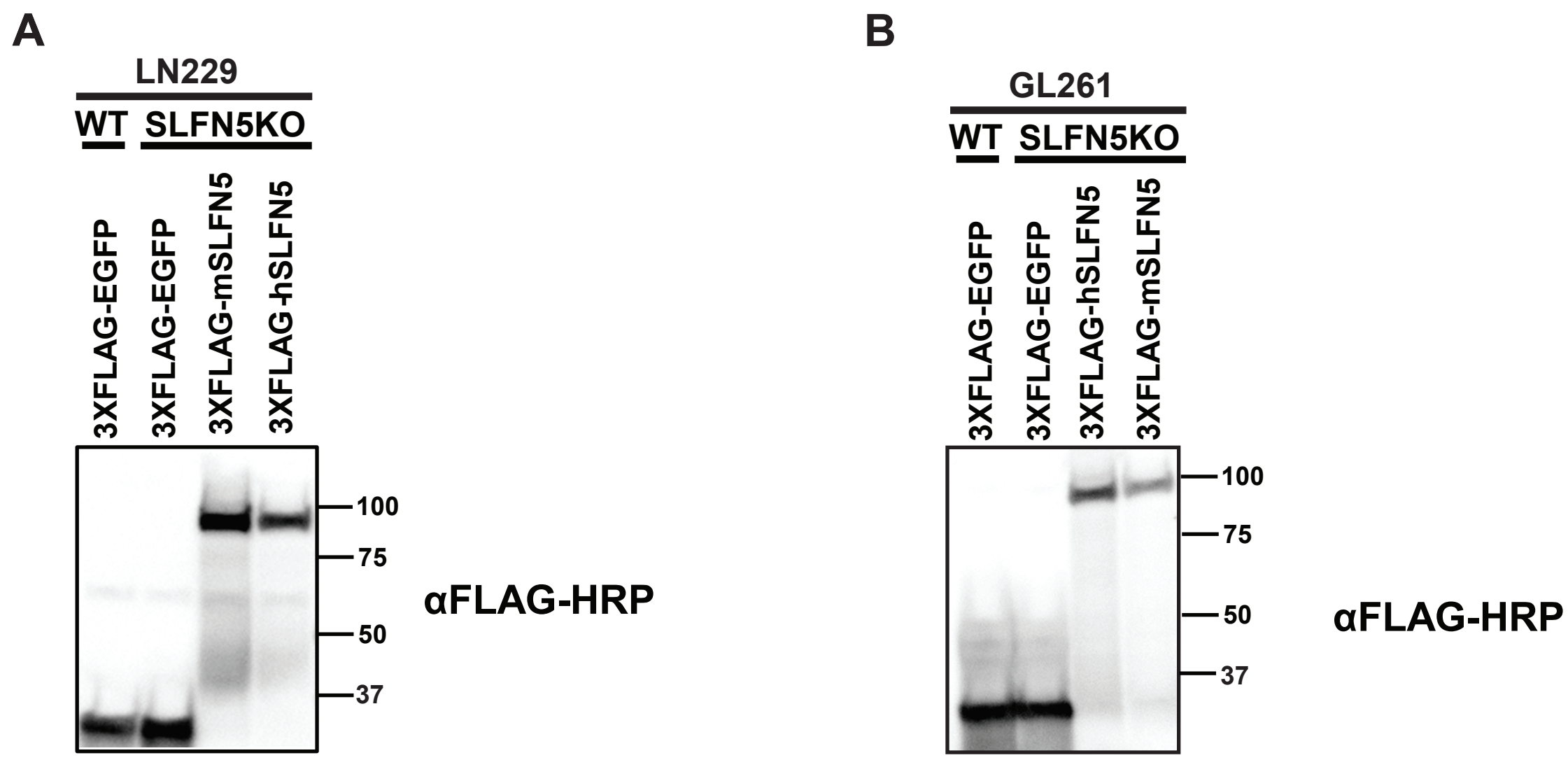

Supplementary Figure 2: Expression of human and mouse SLFN5 in LN229-SLFN5KO **(A)** or GL261-SLFN5KO **(B)** cells. Wild-type (WT) and SLFN5KO cells were transiently transfected with either 3XFLAG-EGFP, 3XFLAG-hSLFN5, or 3XFLAG-mSLFN5. Following transfection, cells were lysed and processed for immunoblotting. Anti-FLAG-HRP antibody was used to assess expression of FLAG-tagged EGFP, hSLFN5, and mSLFN5.

Supplementary Figure 3

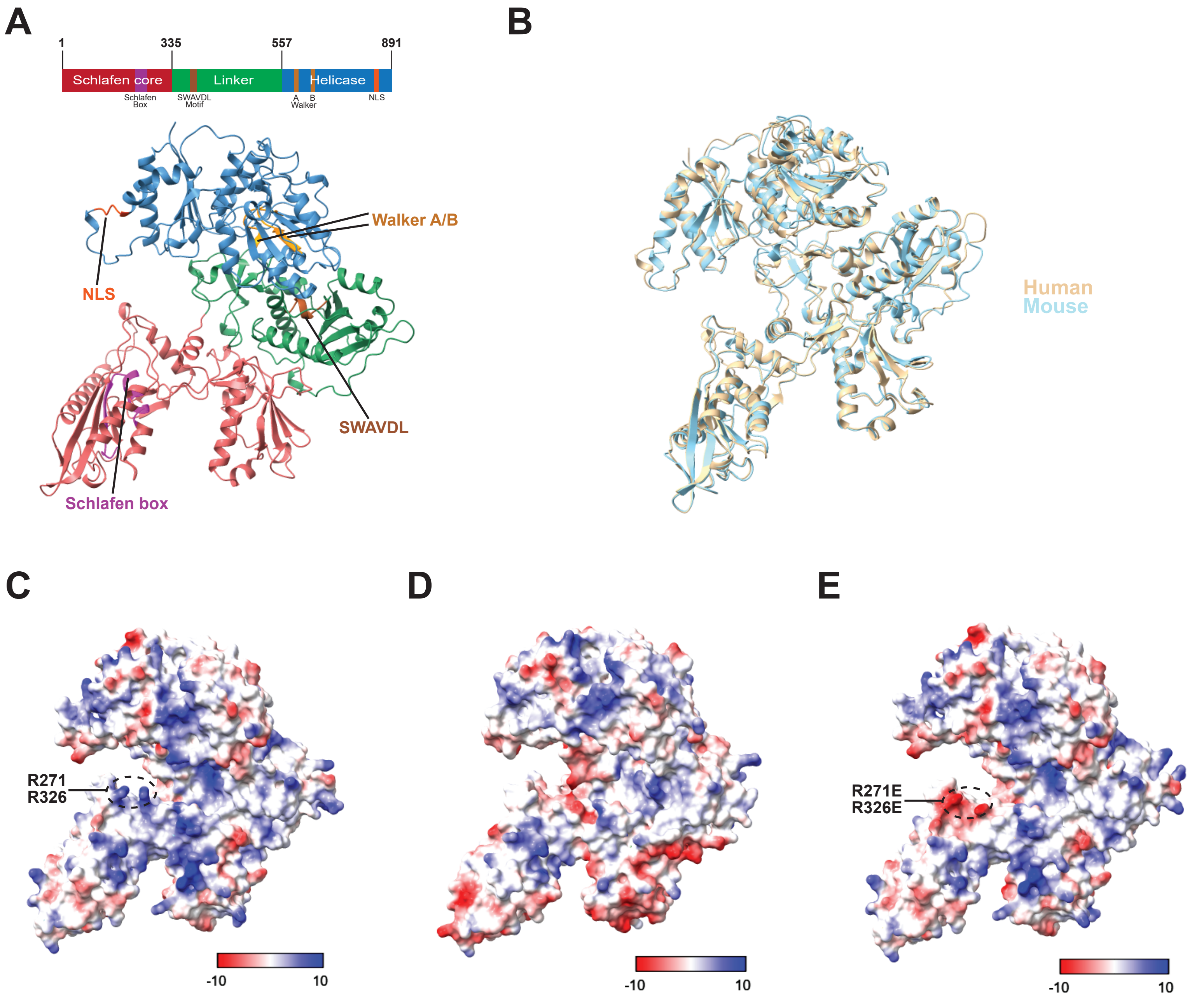

Supplementary Figure 3. Structure of human and mouse SLFN5. (A) Domain schematic of SLFN5 architecture and a ribbon representation of AlphaFold4 model prediction of human SLFN5 depicting the Schlafen box in pink, the SWAVDL motif in brown, the Walker A/B motifs in yellow and the nuclear localization signal (NLS) in orange (modeled using AlphaFold4: AF-Q08AF3-F1-v6). **(B)** AlphaFold4 prediction of human (depicted in gold) and mouse (depicted in sky blue) SLFN5 superimposed using UCSF ChimeraX (AF-Q08AF3-F1-v6 for human; AF-Q8CBA2-F1-v6 for mouse). **(C-E)** Electrostatic potential depiction of wild-type human SLFN5 **(C)**, wild-type mouse SLFN5 **(D)**, and human SLFN5 containing R271E and R326E mutations **(E)** calculated using UCSF ChimeraX.

Supplementary Figure 4

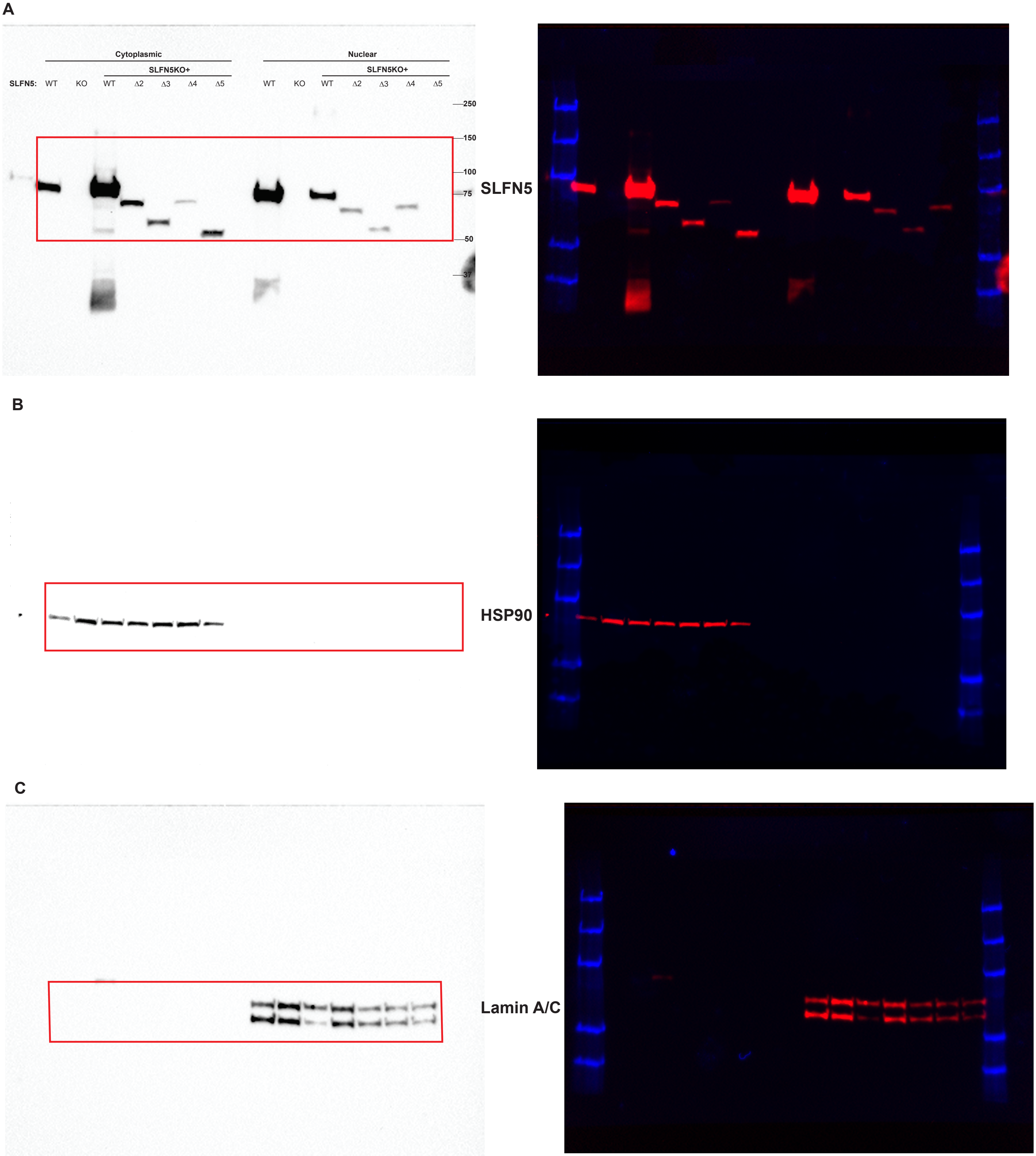

Supplementary Figure 4. Uncropped western blots related to Figure 1B. Immunoblot for SLFN5 **(A)**, HSP90 **(B)** and Lamin A/C **(C)**.

Supplementary Figure 5

A

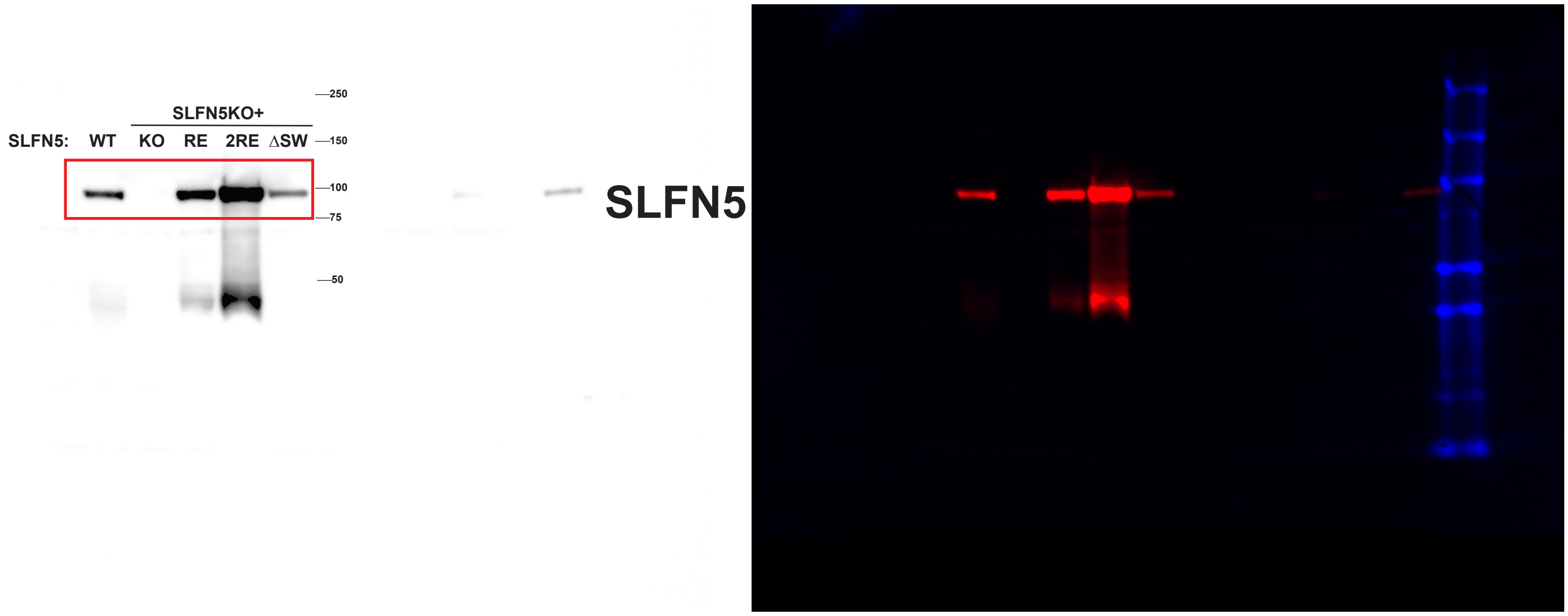

B

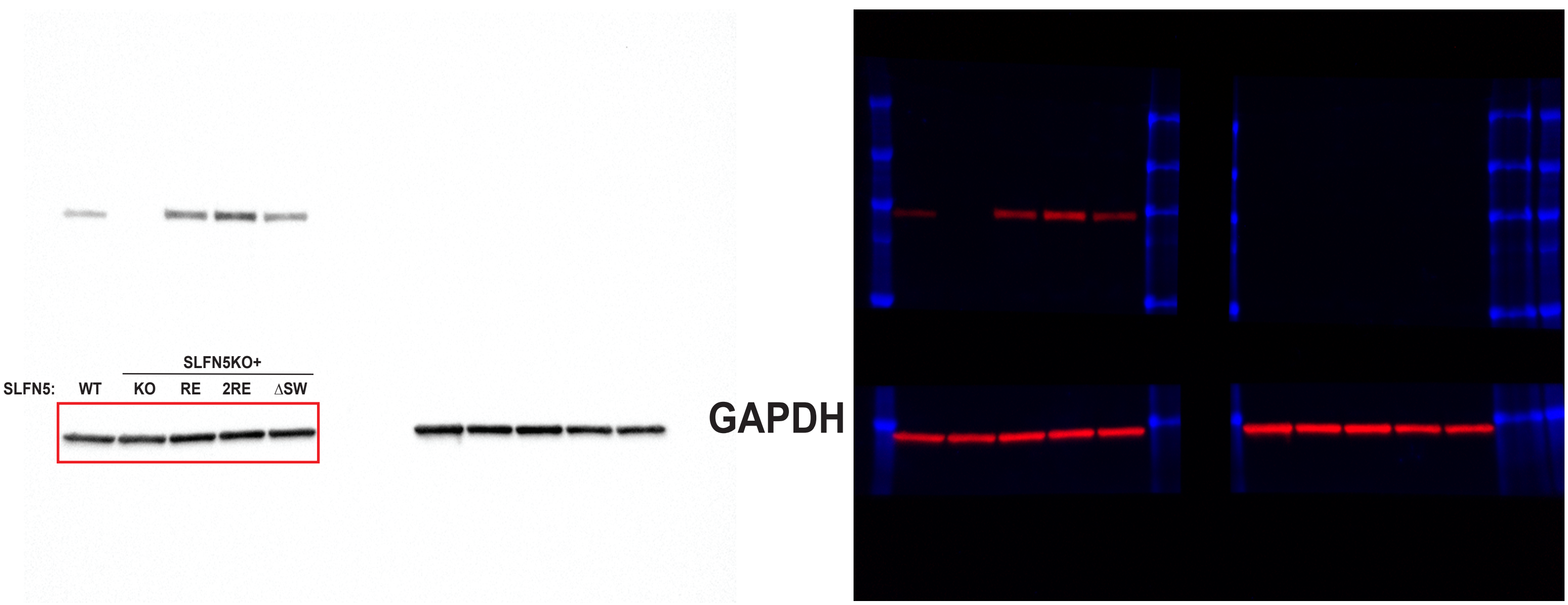

Supplementary Figure 5. Uncropped western blots related to Figure 3B. Immunoblot for SLFN5 **(A)** and GAPDH **(B)**.

Supplementary Figure 6

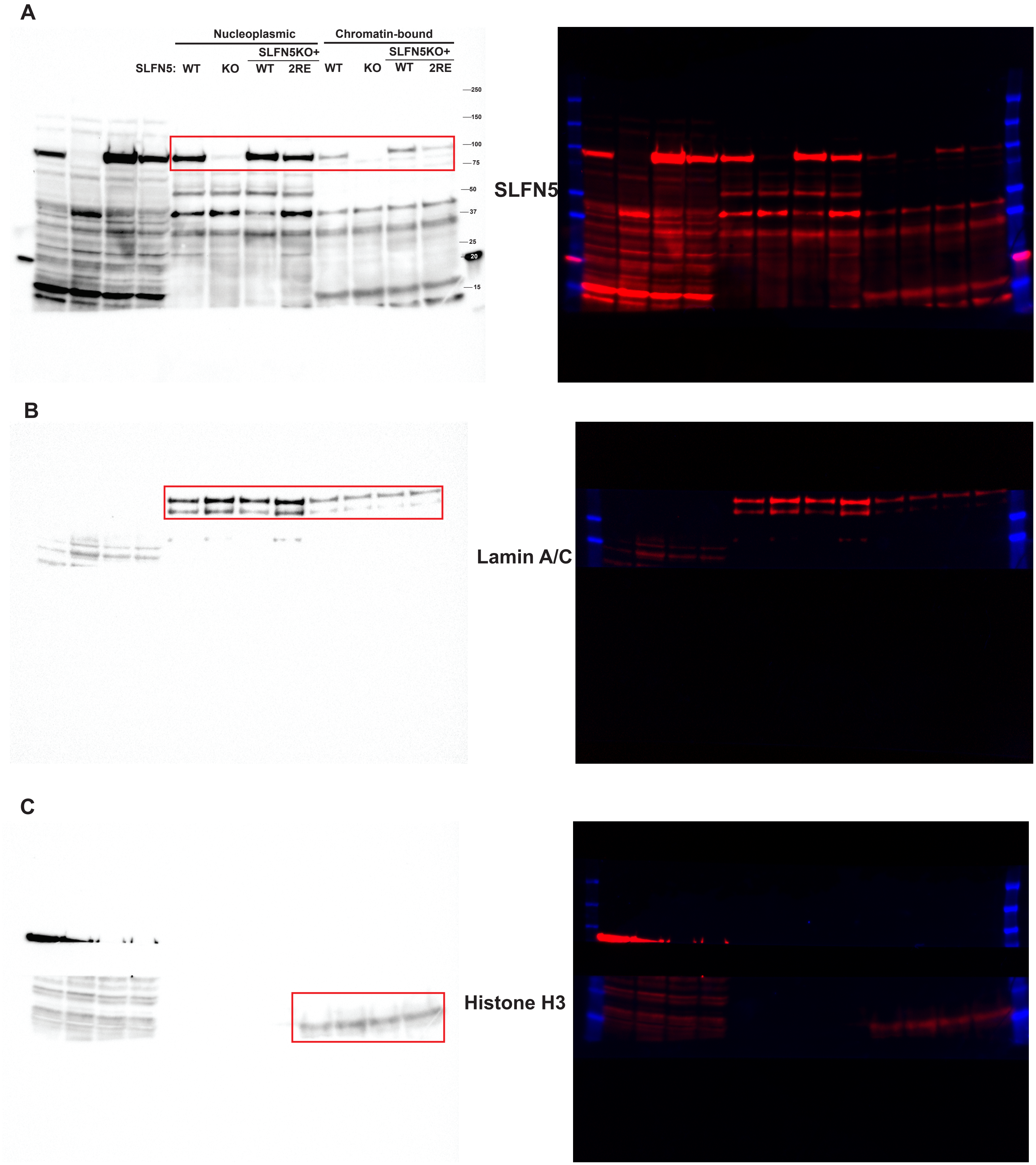

Supplementary Figure 6. Uncropped western blots related to Figure 4A. Immunoblot for SLFN5 **(A)**, Lamin A/C **(B)**, and Histone H3 **(C)**.

Supplementary Table 1

Reagent/Resource	Reference or Source	Identifier or Catalog Number
**Experimental Models**		
LN229	ATCC	CRL-2611
LN229-SLFN5KO	This study	
LN229-SLFN5KO+SLFN5WT	This study	
LN229-SLFN5KO+SLFN5Δ2	This study	
LN229-SLFN5KO+SLFN5Δ3	This study	
LN229-SLFN5KO+SLFN5Δ4	This study	
LN229-SLFN5KO+SLFN5Δ5	This study	
LN229-SLFN5KO+SLFN5R271E	This study	
LN229-SLFN5KO+SLFN5R271E/R326E	This study	
LN229-SLFN5KO+SLFN5ΔSWAVDL	This study	
Lenti-X 293T	Clontech	632180
GL261	Maciej Lesniak<br>Northwestern University	CVCL_Y003
GL261-SLFN5KO	This study	
**Recombinant DNA**		
Lenti-X Packaging Single Shots (VSV-G)	Clontech	631275
SLFN5 CRISPR/Cas9 KO Plasmid (h2)	Santa Cruz	sc-408333-KO-2
SLFN5 HDR Plasmid (h2)	Santa Cruz	sc-408333-HDR-2

Slfn5 CRISPR/Cas9 KO Plasmid (m)	Santa Cruz	sc-435875
Slfn5 HDR Plasmid (m)	Santa Cruz	sc-435875-HDR
pGF1-ISRE-Luc	PMCID: PMC5821504	
pRL-TK	Promega	E2241
pEZ-M12-3XFlag-SLFN5 (h)	GeneCopoeia	EX-H0940-M12
pEZ-M12-3XFlag-SLFN5(m)	GeneCopoeia	EX-Mm36296-M12
pEZ-M12-3XFlag-EGFP	GeneCopoeia	EX-EGFP-M12
pENTR4-Flag (w210-2)	Addgene	Addgene_17423
pLenti CMV Hygro DEST (w117-1)	Addgene	Addgene_17454
**Antibodies**		
Rabbit anti-SLFN5	Sigma-Aldrich	AB_2189993
Mouse anti-GAPDH	Millipore	AB_2107445
Mouse anti-Flag-M2-HRP	Sigma-Aldrich	AB_439702µ
Rabbit anti-Lamin A/C	Cell Signaling Technology	AB_2136278
Mouse anti-Histone H3	Cell Signaling Technology	AB_2756816
Normal Rabbit IgG	Cell Signaling Technology	AB_1031062
Anti-Rabbit IgG, HRP-linked Antibody	Cell Signaling Technology	AB_2099233
Goat anti-Mouse IgG (H+L)-HRP Conjugate	Bio-Rad	AB_11125547
Goat anti-Rabbit IgG (H+L) Highly Cross-Absorbed Secondary Antibody, Alexa Fluor 488	Thermo Fisher	AB_2576217
**Oligonucleotides and other sequence-based reagents**		

BamHI-SLFN5 FOR Primer	IDT	5'-GGCCGGATCCAGTCTTAGGATTGATGTG-3'
KpnI-SLFN5 REV Primer	IDT	5'-GGCCGGTACCTCACACAGAAGCCTTCAG-3'
SLFN5 del190-305 (SLFN5Δ2) FOR Primer	IDT	5'-GTGTTTGCCAAAGTGCCT-3'
SLFN5 del190-305 (SLFN5Δ2) REV Primer	IDT	5'-AGGAAGGTTGAGTTTTTCC-3'
SLFN5 del305-574 (SLFN5Δ3) FOR Primer	IDT	5'-TTGTTTGTTCATGGCTTACC-3'
SLFN5 del305-574 (SLFN5Δ3) REV Primer	IDT	5'-CACCGCACAGCAGAATTT-3'
SLFN5 del574-690 (SLFN5Δ4) FOR Primer	IDT	5'-CACTTGAGTTGCAGTGGC-3'
SLFN5 del574-690 (SLFN5Δ4) REV Primer	IDT	5'-CAACTCTCTGGTCTTGCG-3'
SLFN5 del557-891 (SLFN5Δ5) FOR Primer	IDT	5'-TGAGGTACCGGTACCGAA-3'
SLFN5 del557-891 (SLFN5Δ5) REV Primer	IDT	5'-CAGTAGGTTCAAAACCTCAGAG-3'
SLFN5 R271E FOR Primer	IDT	5'-CTGCACACAG**gag**CCTGAGATAAAATATG-3'
SLFN5 R271E REV Primer	IDT	5'-AAATGATGGACAGGTAGC-3'
SLFN5 R326E FOR Primer	IDT	5'-ATTGCCCACA**gag**GAATGGACTGCTTG-3'
SLFN5 R326E REV Primer	IDT	5'-TGTCTCACACGGTTGTCC-3'

SLFN5 delSWAVDL (SLFN5ΔSW) FOR Primer	IDT	5'-GGTCTGCAAGAGAAGCAG-3'
SLFN5 delSWAVDL (SLFN5ΔSW) REV Primer	IDT	5'-TTGAGAAAAAATCAATATTCCTTGAG-3'
ChIP *ISG15* FOR Primer	IDT	5'-CCACTTTTGCTTTTCCCTGTC-3'
ChIP *ISG15* REV Primer	IDT	5'-AGTTTCGGTTTCCCTTTCCC-3'
ChIP *OAS2* FOR Primer	IDT	5’-CGCTGCAGTGGGTGGAGAGA-3’
ChIP *OAS2* REV Primer	IDT	5’-GCCGGCAAGACAGTGAATGG-3’
ChIP *MX1* FOR Primer	IDT	5'-GGGACAGGCATCAACAAAGCC-3'
ChIP *MX1* REV Primer	IDT	5'-GCCCTCTCTTCTTCCAGGCAAC-3'
ISG15 TaqMan Primer	Thermo Fisher	Hs01921425_s1
MX1 TaqMan Primer	Thermo Fisher	Hs00895608_m1
IRF7 TaqMan Primer	Thermo Fisher	Hs01014809_g1
CD274 TaqMan Primer	Thermo Fisher	Hs00204257_m1
PDCD1LG2 TaqMan Primer	Thermo Fisher	Hs00228839_m1
GAPDH TaqMan Primer	Thermo Fisher	Hs03929097_g1
Slfn5 TaqMan Primer	Thermo Fisher	Mm04335928_m1
Isg15 TaqMan Primer	Thermo Fisher	Mm01705338_s1
Mx1 TaqMan Primer	Thermo Fisher	Mm01218004_m1

Irf7 TaqMan Primer	Thermo Fisher	Mm00516793_g1
Cd274 TaqMan Primer	Thermo Fisher	Mm00452054_m1
Pdcd1lg2 TaqMan Primer	Thermo Fisher	Mm00451734_m1
Gapdh TaqMan Primer	Thermo Fisher	Mm99999915_g1
**Chemicals, Enzymes and other reagents**		
BamHI-HF	New England Biolabs	R3136S
KpnI-HF	New England Biolabs	R3142S
IFNα (human)	Infergen	
IFNα (mouse)	Miltenyi Biotec	130-134-225
Puromycin	Thermo Fisher	A11138-03
Hygromycin B (50 mg/mL)	Thermo Fisher	10687010
DMEM	Thermo Fisher	11965118
Opti-MEM	Thermo Fisher	31985-070
Turbofect Transfection Reagent	Thermo Fisher	R0531
TransDux Max Lentivirus Transduction Enhancer	System Biosciences	LV860A-1
Lenti-X GoStick	Clontech	631280
Lenti-X Concentrator	Clontech	631231
ProLong™ Diamond Antifade Mountant with DAPI	Thermo Fisher	P36962
Clarity™ Western ECL Substrate	Bio-Rad	170-5061
PowerTrack™ SYBR™ Green Master Mix	Thermo Fisher	A46109
TaqMan™ Fast Advanced Master Mix	Thermo Fisher	4444557
Dual-Luciferase® Reporter Assay System	Promega	E1910

**Software**		
Prism v9.0	Graphpad	SCR_002798
Excel	Microsoft	SCR_016137
Image Lab	Bio-Rad	SCR_014210
Adobe Photoshop	Adobe	SCR_014199
Adobe Illustrator	Adobe	SCR_010279
NIS-Elements	Nikon	SCR_014329
AlphFold Protein Structure Database	https://alphafold.ebi.ac.uk/	SCR_023662
AlphaFold4	https://alphafoldserver.com/welcome	SCR_025885
ChimeraX 1.11	UCSF	SCR_015872
**Other**		
Rneasy Plus Mini Kit	Qiagen	74136
High Capacity cDNA Reverse Transcription Kit	Thermo Fisher	4368814
SimpleChIP® Enzymatic Chromatin IP Kit (Magnetic Beads)	Cell Signaling Technology	9003
Q5® Site-Directed Mutagenesis Kit	New England Biolabs	E0554S
NE-PER™ Nuclear and Cytoplasmic Extraction Reagent Kit	Thermo Fisher	78833
Subcellular Protein Fractionation Kit for Cultured Cells	Thermo Fisher	78840
