# Supplementary material for: SLFN5 restrains type I interferon responses and promotes glioblastoma via its N-terminal Schlafen core domain
Source: Commun Biol. 2026 May 27;9:1128. doi: 10.1038/s42003-026-10378-7 (PMC13493812; doi:10.1038/s42003-026-10378-7)
Supplement: Supplementary file 2 [file 42003_2026_10378_MOESM2_ESM.pdf]

Description of Additional Supplementary files

File name: Supplementary Data

Description: The data that support the findings of this study are available in the main text or the supplemental materials; values for all data points in graphs are reported in the Supplementary Data.
